# Deciphering the Role of the rs2651899, rs10166942, and rs11172113 Polymorphisms in Migraine: A Meta-Analysis

**DOI:** 10.3390/medicina58040491

**Published:** 2022-03-29

**Authors:** Vasileios Siokas, Ioannis Liampas, Athina-Maria Aloizou, Maria Papasavva, Christos Bakirtzis, Eleftherios Lavdas, Panagiotis Liakos, Nikolaos Drakoulis, Dimitrios P. Bogdanos, Efthimios Dardiotis

**Affiliations:** 1 Laboratory of Neurogenetics, Department of Neurology, University Hospital of Larissa, Faculty of Medicine, School of Health Sciences, University of Thessaly, 41100 Larissa, Greece; vsiokas@med.uth.gr (V.S.); liampasioannes@gmail.com (I.L.); aaloizou@med.uth.gr (A.-M.A.); 2 Research Group of Clinical Pharmacology and Pharmacogenomics, Faculty of Pharmacy, School of Health Sciences, National and Kapodistrian University of Athens, Panepistimiopolis Zografou, 15771 Athens, Greece; mariapapa@pharm.uoa.gr (M.P.); drakoulis@pharm.uoa.gr (N.D.); 3 B’ Department of Neurology, AHEPA University Hospital, Aristotle University of Thessaloniki, 54636 Thessaloniki, Greece; bakirtzischristos@yahoo.gr; 4 Department of Biomedical Sciences, University of West Attica, 12243 Athens, Greece; llavdas@uniwa.gr; 5 Department of Medical Imaging, Animus Kyanoys Larisas Hospital, 41222 Larissa, Greece; 6 Laboratory of Biochemistry, Faculty of Medicine, University of Thessaly, 41100 Larissa, Greece; pliakos@med.uth.gr; 7 Department of Rheumatology and clinical Immunology, University General Hospital of Larissa, Faculty of Medicine, School of Health Sciences, University of Thessaly, Viopolis, 40500 Larissa, Greece; bogdanos@uth.gr

**Keywords:** migraine, headache, rs2651899, rs10166942, rs11172113, genetics, polymorphism, PRDM16, TRPM8, LPR1

## Abstract

The genetic basis of migraine is rather complex. The rs2651899 in the PR/SET domain 16 (PRDM16) gene, the rs10166942 near the transient receptor potential cation channel subfamily M member 8 (TRPM8) gene, and the rs11172113 in the LDL receptor-related protein 1 (LRP1) gene, have been associated with migraine in a genome-wide association study (GWAS). However, data from subsequent studies examining the role of these variants and their relationship with migraine remain inconclusive. The aim of the present study was to meta-analyze the published data assessing the role of these polymorphisms in migraine, migraine with aura (MA), and migraine without aura (MO). We performed a search in the PubMed, Scopus, Web of Science, and Public Health Genomics and Precision Health Knowledge Base (v7.7) databases. In total, eight, six, and six studies were included in the quantitative analysis, for the rs2651899, rs10166942, and rs11172113, respectively. Cochran’s Q and I2 tests were used to calculate the heterogeneity. The random effects (RE) model was applied when high heterogeneity was observed; otherwise, the fixed effects (FE) model was applied. The odds ratios (ORs) and the respective 95% confidence intervals (CIs) were calculated to estimate the effect of each variant on migraine. Funnel plots were created to graphically assess publication bias. A significant association was revealed for the CC genotype of the rs2651899, with the overall migraine group (RE model OR: 1.32; 95% CI: 1.02–1.73; *p*-value = 0.04) and the MA subgroup (FE model OR: 1.40; 95% CI: 1.12–1.74; *p*-value = 0.003). The rs10166942 CT genotype was associated with increased migraine risk (FE model OR: 1.36; 95% CI: 1.18–1.57; *p*-value < 0.0001) and increased MO risk (FE model OR: 1.41; 95% CI: 1.17–1.69; *p*-value = 0.0003). No association was detected for the rs11172113. The rs2651899 and the rs10166942 have an effect on migraine. Larger studies are needed to dissect the role of these variants in migraine.

## 1. Introduction

Migraine is a complex disorder of the brain, with great variety in its pathogenesis, clinical presentation, genetic make-up, and therapeutic approach [1]. It is the second most common cephalalgia, after the tension-type headache [2]. Moreover, it is considered to be among the commonest neurological disorders globally, while it confers greater disability compared with other neurological diseases [3].

Phenotypically, migraine manifests with recurrent episodes characterized by pulsating intense pain in the head unilaterally [4]. Additionally, symptoms such as photophobia, vomiting, phonophobia, and nausea usually accompany migraine attacks [5]. Migraine with aura (MA) and migraine without aura (MO) are considered to be the major migraine subtypes, which are mainly differentiated by the presence of focal neurological symptoms that can either precede or accompany headache in patients with MA [6].

From a pathophysiological perspective, several theories including various molecular mechanisms have been connected to migraine risk, such as the release of vasoactive neuropeptides vascular dysfunction, vasodilation, defective function of brain networks, plasma protein extravasation, cortical spreading depression (CSD), and “neurogenic inflammation” [7,8]. Moreover, increased glucose uptake has been reported in patients, with migraine especially in the posterior white matter of the cerebrum and cerebellum [9]. Additionally, glucose levels and metabolism may influence the frequency of CSD, and as such, migraine development [10,11].

While the pathophysiological pathways via which the previously referred mechanisms can lead to the migraine are not fully understood [12], there are multiple lines of evidence that genetic, environmental, and epigenetic factors all contribute, to some extent, to migraine’s susceptibility [13,14]. Among the environmental factors, body mass index (BMI), smoking, dietary habits, nutrients, physical activity, and socioeconomic status (to name a few) have been incriminated in altering migraine risk or as precipitating factors for migraine attacks [15,16,17,18,19,20,21].

The genetic architecture of migraine is complex, as migraine is considered a polygenic disease, where a few genetic factors are implicated in its appearance and phenotypic traits [22,23,24]. The complexity of the genetic influence on migraine is also evident considering that triggers and factors (genetic and environmental) heavily vary amongst the affected patients [25]. Nevertheless, there are a few known mutations in single genes that can cause the entity known as the familial hemiplegic migraine (FHM) [26]. As such, mutations in the Calcium Voltage-Gated Channel Subunit Alpha1 A (CACNA1A), encoding the α1 subunit of the brain specific P/Q- type calcium channel, in ATPase Na^+^/K^+^ Transporting Subunit Alpha 2 (ATP1A2), encoding the sodium–potassium- transporting ATPase, in Sodium Voltage-Gated Channel Alpha Subunit 1 (SCN1A) encoding a voltage- gated sodium channel subunit, can all lead to FHM [26,27]. Additionally, other monogenetic migraine with aura syndromes such as cerebral autosomal dominant arteriopathy with subcortical infarcts and leukoencephalopathy (CADASIL), retinal vasculopathy with cerebral leukoencephalopathy and systemic manifestations (RVCL-S), and familial advanced sleep phase syndrome (FASPS), also exists [23]. However, phenotypic appearance may exhibit great variance (with the migraine not being a prominent feature), and there are also cases where novel mutations have been identified [27].

Apart from FHM and monogenic migraine with aura syndromes, there are polymorphisms (e.g., the MTHFR C677T, and BDNF rs6265 gene polymorphisms) that have been further found to be associated with migraine [28,29,30,31,32] and other headaches [33,34]. In 2011, in a genome-wide association study (GWAS), three genetic loci emerged as genetic risk factors for migraine [35]. These genetic loci are the rs2651899 in the PR/SET domain 16 (PRDM16) gene, the rs10166942 near the transient receptor potential cation channel subfamily M member 8 (TRPM8) gene, and the rs11172113 in the LDL receptor-related protein 1 (LRP1) gene [35]. These results for the rs10166942 and the rs11172113 have been replicated by a further GWAS [36]. However, results from subsequent studies examining the role of the aforementioned variants and their relationship with migraine remain inconclusive. While the PRDM16 rs2651899 has been reported to associate with migraine and MA and/or MO subtypes in an earlier meta-analysis [37], studies that followed revealed no association with migraine [38], while both the alleles of the polymorphism have been associated with increased migraine risk [39,40]. In the same manner, studies for the rs10166942 near the TRPM8 gene and the LRP1 rs11172113 have yielded inconsistent results [41].

In view of the former considerations, the aim of the present study was to retrieve, review, and meta-analyze the available published data assessing the role of the PRDM16 rs2651899, the rs10166942 near the TRPM8 gene, and the LPR1 rs11172113 polymorphisms on migraine. Additionally, we attempted to assess the role of these variants on the risk of the main migraine endophenotypes, namely the MA and the MO.

## 2. Materials and Methods

### 2.1. General Information

The Preferred Reporting items Systematic Reviews and Meta-Analyses (PRISMA) guidelines (Supplementary File S1) were applied for the current meta-analysis [42], while this study was not registered in any database. Two authors (V.S. and I.L.), independently performed the processes, while any divergences were unraveled by a third author (E.D.).

### 2.2. Literature Search Strategy

We searched through the PubMed, Scopus, Web of Science, and Public Health Genomics and Precision Health Knowledge Base (v7.7) databases for eligible articles examining the relationship between migraine and the PRDM16 rs2651899, the rs10166942 near the TRPM8 gene, and the LPR1 rs11172113 polymorphisms (the last search was performed on 18 March 2022). The search for each variant was performed separately. We used the term “migraine” in combination with either “rs2651899”, or “rs10166942”, or “rs11172113”, as free words. The PubMed algorithm of the literature search for the present meta-analysis is presented at Supplementary File S2.

### 2.3. Identification of Eligible Articles

We initially checked titles and abstracts of identified articles for relevance. From the articles that passed the initial screening, full texts were retrieved. Additionally, the reference lists of the identified articles were scanned for supplementary eligible studies.

### 2.4. Eligibility Criteria

Studies that met the following criteria were included: (1) written in the English language, (2) publication before the 18 March 2022, and (3) the absolute genotype numbers for the examined variants were available for controls and patients with migraine. Data from GWASs and studies containing irrelevant data were not included.

### 2.5. Data Extraction

The following data from each eligible study were extracted when possible: (1) author, (2) year of publication, (3) ethnicity/location of the examined population, (4) numbers (n) of cases with migraine and controls, (5) age at onset of migraine, (6) mean age of the participants during examination, (7) number of males and females in patients with migraine and controls, (8) criteria applied to the assessment of the diagnosis of migraine, (9) test of the Hardy–Weinberg Equilibrium (HWE) principle, (10) applied method for correction for multiple comparisons, (11) genotype absolute numbers, and (12) main results.

### 2.6. Statistical Analysis

#### 2.6.1. Calculation of the Effect Size

Statistical analyses were performed with Review Manager (RevMan) Version 5.4 (https://training.cochrane.org/online-learning/core-software-cochrane-reviews/revman/revman-5-download, accessed on 16 December 2021). The odds ratios (ORs) and the respective 95% confidence intervals (CIs) were calculated in order for the effect of each variant on migraine to be estimated. The following effects were calculated: (a) homozygosity for the variant allele genotype, (b) heterozygosity, and (c) homozygosity for the wild-type allele. Three phenotypic traits were considered as outcomes: (a) migraine, (b) MA, and (c) MO. Statistically significant values were considered those with values lower than 0.05 (*p* < 0.05).

#### 2.6.2. Heterogeneity and Assessment of Publication Bias

Cochran’s Q and I2 tests were used to calculate the heterogeneity. The random effects (RE) model was applied when high heterogeneity was observed (P_Q_ < 0.10 and/or I^2^ > 75%) [43]; otherwise, the fixed effects (FE) model was applied [44]. Funnel plots were created to graphically assess the publication bias.

## 3. Results

### 3.1. Study Selection and Study Characteristics

#### 3.1.1. PRDM16 rs2651899

The search of the databases (after the removal of duplicate records) yielded 14 articles, published between 2011 and 2021. After the initial evaluation of titles and abstracts, three articles were excluded as review articles. Consequently 11 full texts were examined for eligibility. Four articles [35,38,45,46] were excluded (GWAS or no available genotypic data). One additional eligible study was identified via the manual screening of the reference lists [47]. Thus, eight studies were finally included in the quantitative meta-analysis [39,40,41,47,48,49,50,51], consisting of 2320 patients with migraine and 2615 controls. The flowchart with the selection procedure of eligible studies for the PRDM16 rs2651899 is presented in Supplementary File S3. The baseline characteristics of the studies that fulfilled the eligibility criteria are presented in Table 1.

#### 3.1.2. rs10166942 near TRPM8 Gene

The search of the databases (after the remove of duplicate records) yielded 14 articles, published between 2011 and 2021. After the initial evaluation of titles and abstracts, three articles were excluded (no genetic studies or review articles). Consequently 11 full texts were examined for eligibility. Five articles [35,45,46,52,53] were excluded (GWAS or no available genotypic data, no examination of this polymorphism). As such, six studies were finally included in the quantitative meta-analysis [39,40,41,48,50,51], consisting of 1633 patients with migraine and 1514 controls. The flowchart with the selection procedure of eligible studies for the rs10166942 is presented in Supplementary File S4. The baseline characteristics of the studies that fulfilled the eligibility criteria are depicted in Table 1.

#### 3.1.3. LPR1 rs11172113

The search of the databases (after the remove of duplicate records) yielded 18 articles, published between 2011 and 2022. After the initial evaluation of titles and abstracts, four articles were excluded (no genetic studies or review articles). Consequently 14 full texts were examined for eligibility. Eight articles [35,38,45,46,54,55,56,57] were excluded (GWAS or no available genotypic data). Accordingly, six studies were finally included in the quantitative meta-analysis [41,47,48,50,51,58], consisting of 1462 patients with migraine and 1659 controls. The flowchart with the selection procedure of eligible studies for the LPR1 rs11172113 is presented in Supplementary File S5. The baseline characteristics of the studies that fulfilled the eligibility criteria are depicted in Table 1.

**Table 1:** The baseline characteristics of the studies included in the current meta-analysis.

					Cases			Controls			
Author (Year) [Ref]	Population or Location	Gene (Polymorphism)	HWE Test/Multiple Test Correction	Diagnosis Assessment	Mean Age ± SD/Age of Onset ± SD	*n*	Male/Female	Mean Age ± SD	*n*	Male/Female	Main Results and Comments
An et al. (2013) [48]	Han-Chinese	PRDM16 (rs2651899); TRPM8 (rs10166942); and LPR1 (rs11172113)	Yes (cases and controls)/-	International Classification of Headache Disorders, 2nd edition (ICHDII)	36.0 ± 10.9 years/-	207	37/170	35.8 ± 11.5 years	205	49/156	The rs2651899 G allele was associated with migraine and MO in allelic mode. No association for the TRPM8 rs10166942 and the LPR1 rs11172113.
Gosh et al. (2013) [50]	India	PRDM16 (rs2651899); TRPM8 (rs10166942); and LPR1 (rs11172113)	Yes (controls)/Yes (Benjamini–Hochberg false discovery rate (FDR) test)	International Classification of Headache Disorders, 2nd edition (ICHDII)	-/<50 years	340	-	matched	200	matched	Protective effect of the rs2651899 (T) on migraine and MO susceptibility (genotypic, dominant, allelic models). Protective effect of the LPR1 rs11172113 C allele on migraine MA and MO in various models. No association for the TRPM8 rs10166942.
Fan et al. (2014) [51]	Han-Chinese	PRDM16 (rs2651899); TRPM8 (rs10166942); and LPR1 (rs11172113)	Yes (controls)/Yes (Bonferroni)	International Classification of Headache Disorders, 2nd edition (ICHDII)	40.65 ± 12.18 years/24.03 ± 11.13 years	304	53/251	matched	304	matched	The rs2651899 minor allele (C) was associated with migraine and MO. No association for the TRPM8 rs10166942 and the LPR1 rs11172113.
Sintas et al. (2015) [40]	Spanish	PRDM16 (rs2651899); TRPM8 (rs10166942); and LPR1 (rs11172113)	Yes (cases and controls)/10,000 permutations and Bonferroni’s correction	International Classification of Headache Disorders, 2nd edition (ICHDII)	-/13.5 ± 12 years	512	78.13% female	matched	535	78.83% female	The rs2651899 minor allele (C) was nominally associated with migraine and MA. The TRPM8 rs10166942 (T) allele nominally associated with migraine. No significance remained after multiple comparison correction.
An et al. (2017) [47]	Chinese	PRDM16 (rs2651899); and LPR1 (rs11172113)	Yes (controls)/Yes (Benjamini–Hochberg false discovery rate (FDR) test and Bonferroni)	International Classification of Headache Disorders (ICHD-III beta)	-/35.4 ± 10.2 years	581	61/520	34.8 ± 8.9 years	533	57/476	The rs2651899 C allele was associated MO and migraine with family history subgroup. No association for the LPR1 rs11172113.
Ran et al. (2018) [49]	Swedish	PRDM16 (rs2651899);	Yes/-	International Classification of Headache Disorders, 2nd edition (ICHDII)	-	100	-	-	581	56.3% male	No association.
Kaur et al. (2019) [39]	North Indian	PRDM16 (rs2651899) and TRPM8 (rs1016694)	Yes (controls)/-	International Classification of Headache disorders, 3rd edition	35.28 ± 6.6 years/	150	40/110	no statistical difference in terms of age as *p* = 0.35	150	60% females	The rs2651899 T allele was associated with migraine in genotypic, allelic, and dominant model. Association was found for the variant with the MO and the female migraineurs. The TRPM8 rs1016694 was associated with MA and in males.
Kaur et al. (2019) [58]	India	LPR1 (rs11172113)	Yes/-	International Classification of Headache disorders, 3rd edition	MA:35.13 ± 6.0 years/- MO: 36.40 ± 5.2 years/-	100	28/72	34.45 ± 7.6 years	100	38/62	No association
Zafar et al. (2021) [41]	Pakistan	PRDM16 (rs2651899); TRPM8 (rs10166942); and LPR1 (rs11172113	Yes (controls)/-	International Classification of Headache Disorders, 2nd edition (ICHDII)	25.79 ± 5.19 years/-	127	31/96	26.26 ± 5.57 years	120	38/82	The rs2651899 G allele was associated with migraine, MO, and MA. The TRPM8 rs10166942 and the LPR1 rs11172113 were associated with migraine and MO.

PRDM16, PR/SET Domain 16; TRPM8, Transient Receptor Potential Cation Channel Subfamily M Member 8; LRP1, LDL receptor-related protein 1; MA, migraine with aura; MO, migraine without aura; CH, cluster headache.

### 3.2. Tests of Heterogeneity, Effect Size, and Publication Bias

#### 3.2.1. PRDM16 rs2651899

##### Overall Migraine Group

**Figure 1:**
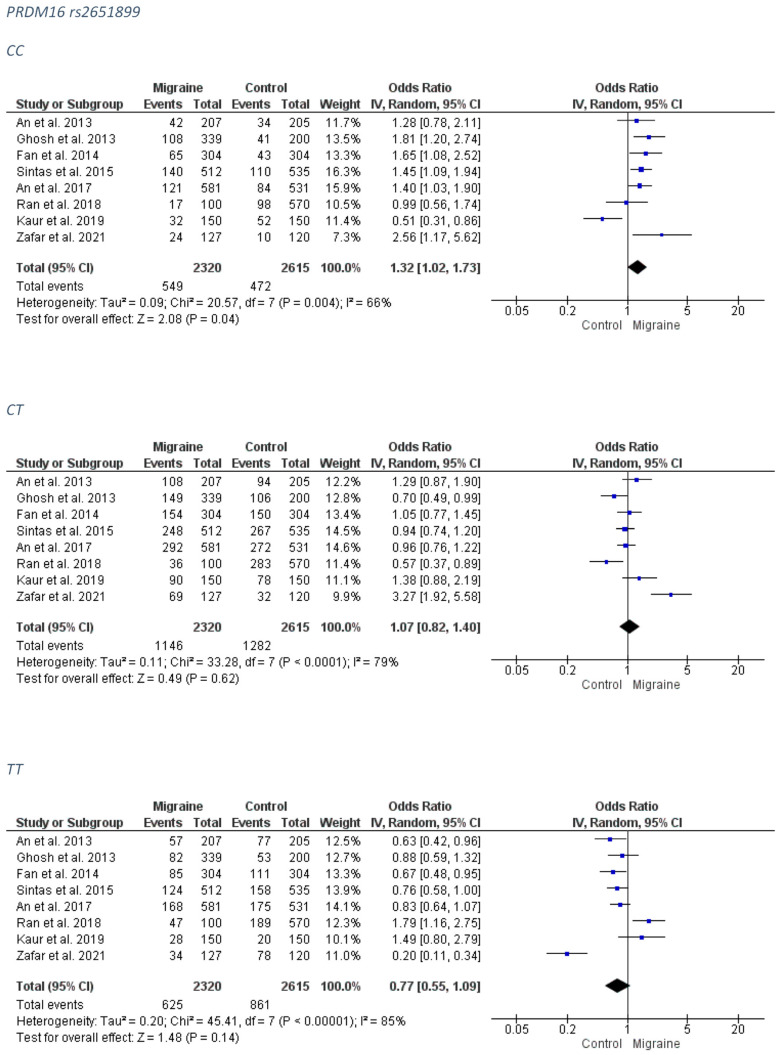
The forest plots presenting the results from meta-analysis of the rs2651899 and overall migraine group.

A significant association was revealed between the PRDM16 rs2651899 and the overall migraine group for the CC genotype (RE model OR: 1.32; 95% CI: 1.02–1.73; *p*-value = 0.04). The forest plots can be accessed in Figure 1. Analysis for publication bias suggested that Zafar et al. [41] overestimated the risk conferring effect of the CT model and oversized the protective impact of the TT model (Supplementary File S6).

##### MA Group

**Figure 2:**
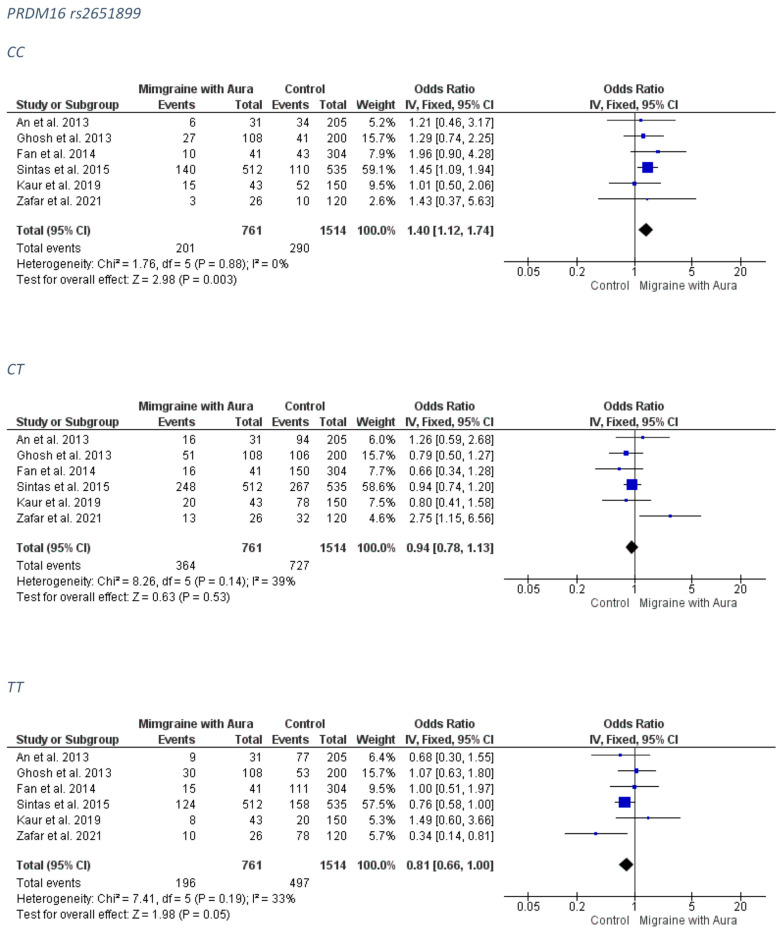
The forest plots presenting the results from meta-analysis of the rs2651899 and migraine with aura group.

A significant association revealed between the PRDM16 rs2651899 and the MA subgroup group for the CC genotype (FE model OR: 1.40; 95% CI: 1.12–1.74; *p*-value = 0.003) and a marginal trend for a protective effect of the AA genotype (FE model OR: 0.81; 95% CI: 0.66–1.00; *p*-value = 0.05). The forest plots can be accessed in Figure 2. Funnel plots were not indicative for publication bias (Supplementary File S7).

##### MO Group

**Figure 3:**
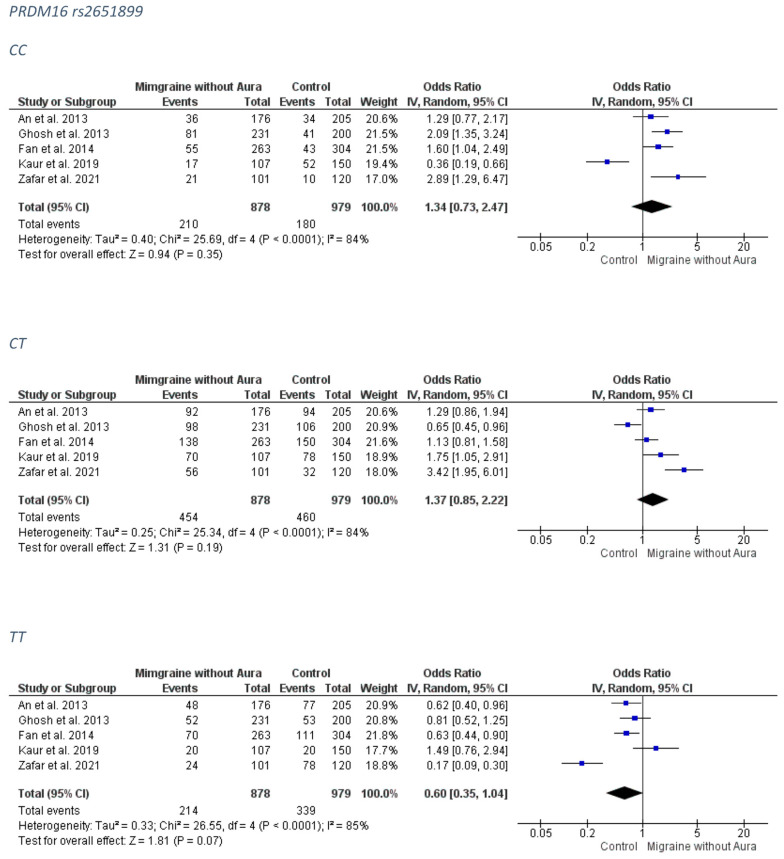
The forest plots presenting the results from meta-analysis of the rs2651899 and migraine without aura group.

No association was revealed for the PRDM16 rs2651899 and the MO subgroup. The forest plots can be accessed in Figure 3. Analysis for publication bias suggested that smaller studies tended to exaggerate the risk conferring association of the CT model (OR probably lies closer to 1.00 than estimated) (Supplementary File S8).

#### 3.2.2. rs10166942 near TRPM8 Gene

##### Overall Migraine Group

**Figure 4:**
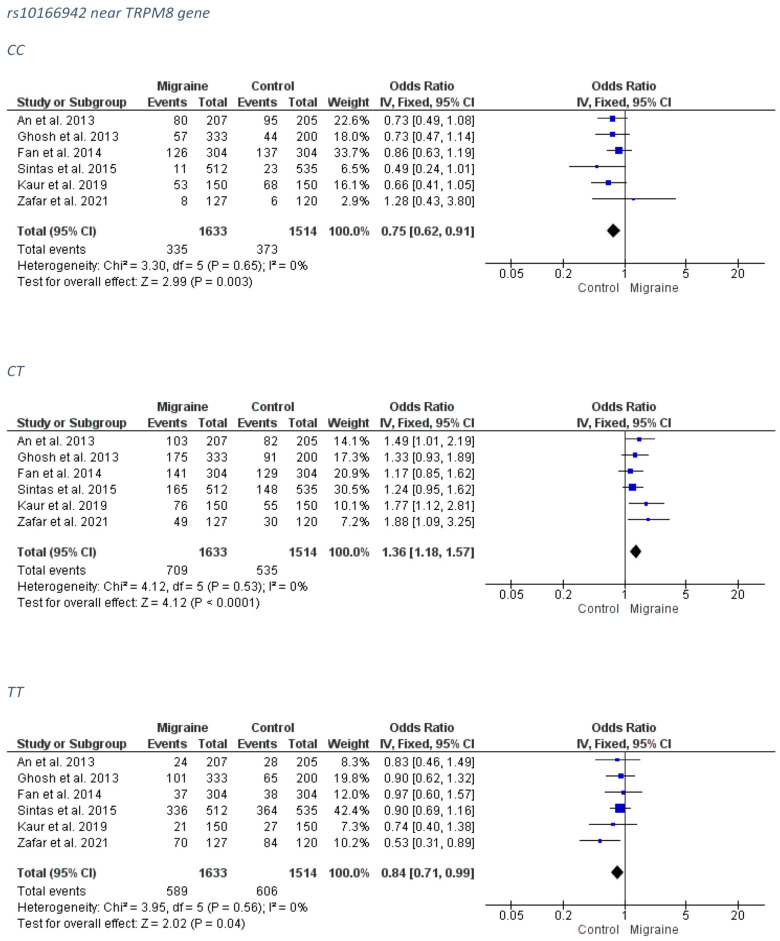
The forest plots presenting the results from meta-analysis of the rs10166942 and overall migraine group.

A significant association for a protective effect was revealed between the rs10166942 and the overall migraine group for the CC genotype (FE model OR: 0.75; 95% CI: 0.62–0.91; *p*-value = 0.003) and for the TT genotype (FE model OR: 0.84; 95% CI: 0.71–0.99; *p*-value = 0.03). On the contrary, the heterozygosity CT was associated with increased migraine risk (FE model OR: 1.36; 95% CI: 1.18–1.57; *p*-value < 0.0001). The forest plots can be accessed in Figure 4. Analysis for publication bias (Supplementary File S9) suggested that smaller, less precise articles appeared to overestimate the risk conferring association of the CT model (the true OR is probably closer to 1.00, i.e., relatively mitigated), as well as the protective effect of the TT model (the true OR may be even equal to 1.00, i.e., no true effect). Publication bias was not apparent with respect to the CC model.

##### MA Group

**Figure 5:**
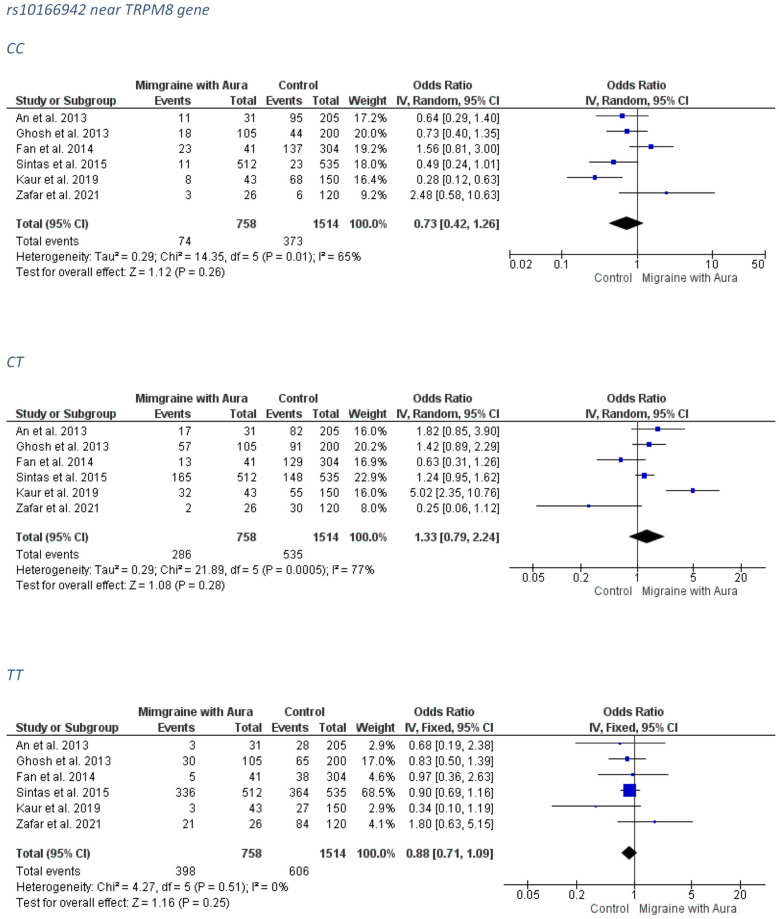
The forest plots presenting the results from meta-analysis of the rs10166942 and migraine with aura group.

No association was detected between the rs10166942 and the MA subgroup. The forest plots can be accessed in Figure 5. The funnel plots are not indicative of a clear direction for a biased publication trend (Supplementary File S10).

##### MO Group

**Figure 6:**
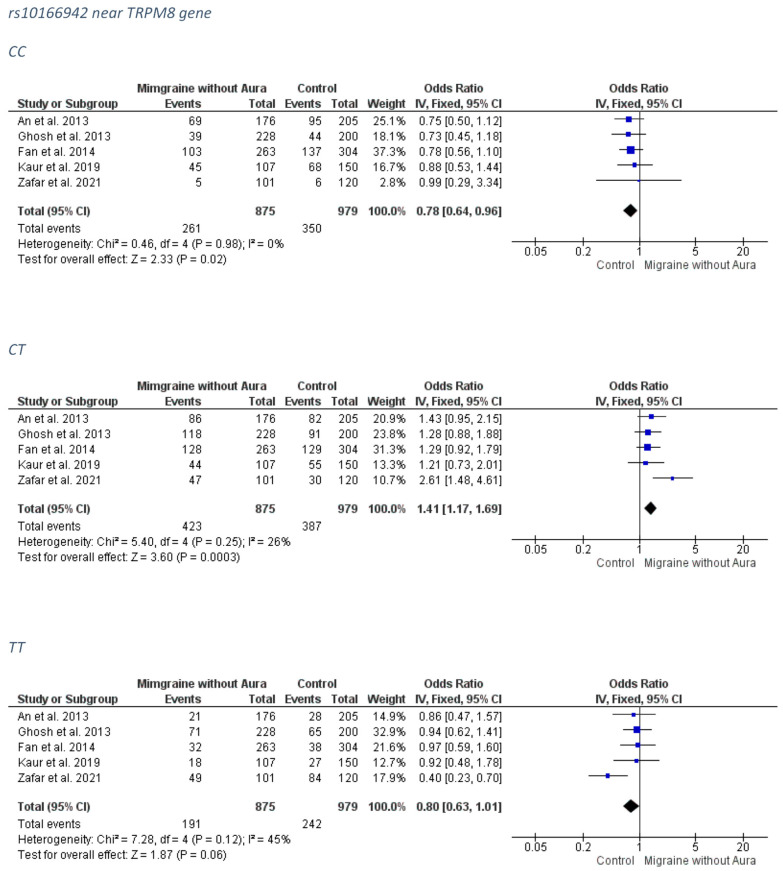
The forest plots presenting the results from meta-analysis of the rs10166942 and migraine without aura group.

A significant association, with a protective effect was revealed between the rs10166942 and the overall migraine group for the CC genotype (FE model OR: 0.78; 95% CI: 0.64–0.96; *p*-value = 0.02) while the heterozygosity CT was associated with increased MO risk (FE model OR: 1.41; 95% CI: 1.17–1.69; *p*-value = 0.0003). A marginal protective effect against MO was found for the TT genotype (FE model OR: 0.80; 95% CI: 0.63–1.01; *p*-value = 0.06). The forest plots can be accessed in Figure 6. Analysis for publication bias (Supplementary File S11) suggested that the smallest, least precise article seemed to mildly downsize the protective effect of the CC model and exaggerate the risk conferring effect of the CT model, as well as the protective effect of the TT model. Therefore, real associations are probably relatively stronger for the CC model and mitigated for the CT and TT models.

#### 3.2.3. LPR1 rs11172113

##### Overall Migraine Group

**Figure 7:**
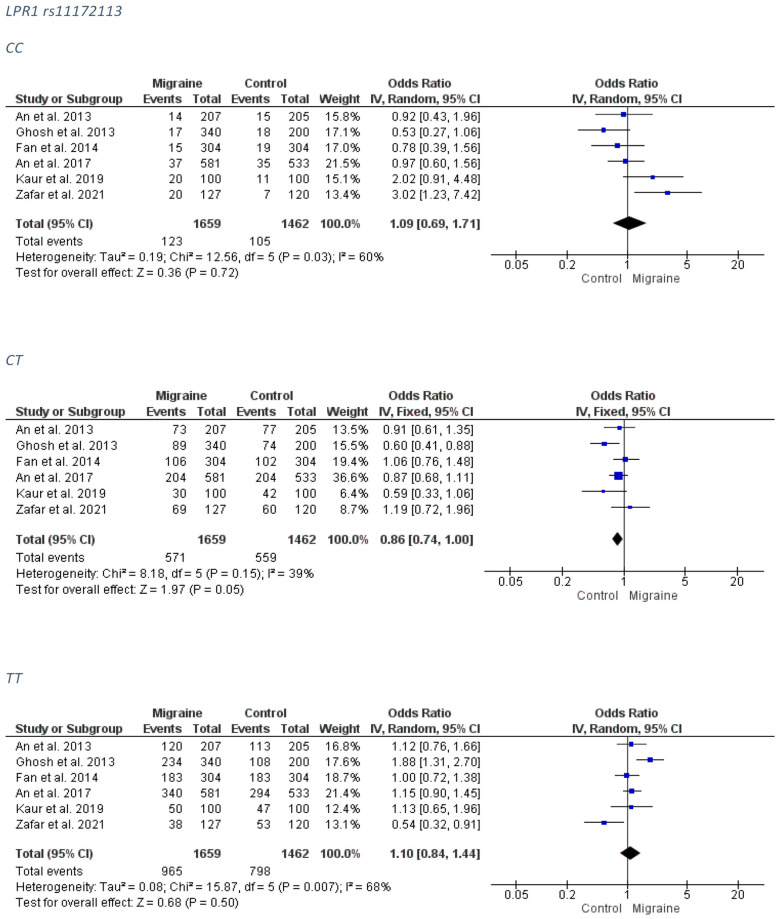
The forest plots presenting the results from meta-analysis of the rs11172113 and overall migraine group.

Only a marginal trend for association was revealed between the LPR1 rs11172113 and migraine for the CT genotype (FE model OR: 0.86; 95% CI: 0.74–1.00; *p*-value = 0.05). The forest plots can be accessed in Figure 7. Analysis for publication bias (Supplementary File S12) suggested that smaller, less precise studies tended to overestimate the true effect (OR) of the CC model, which was probably closer to 1.00 than estimated (i.e., no association), and tended to mildly underestimate the true effect of the recessive model TT. The CT model appears to be less (if at all) affected by publication bias.

##### MA Group

**Figure 8:**
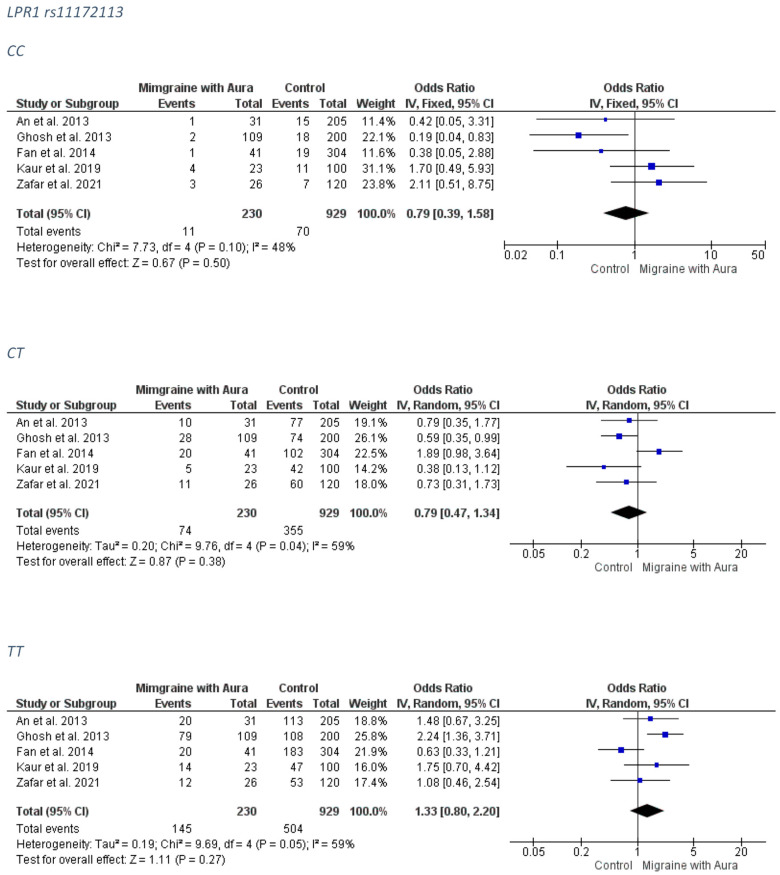
The forest plots presenting the results from meta-analysis of the rs11172113 and migraine with aura group.

No association was detected between the LPR1 rs11172113 and MA subgroup. The forest plots can be accessed in Figure 8. The funnel plots are not indicative of a clear direction for a biased publication trend (Supplementary File S13).

##### MO Group

**Figure 9:**
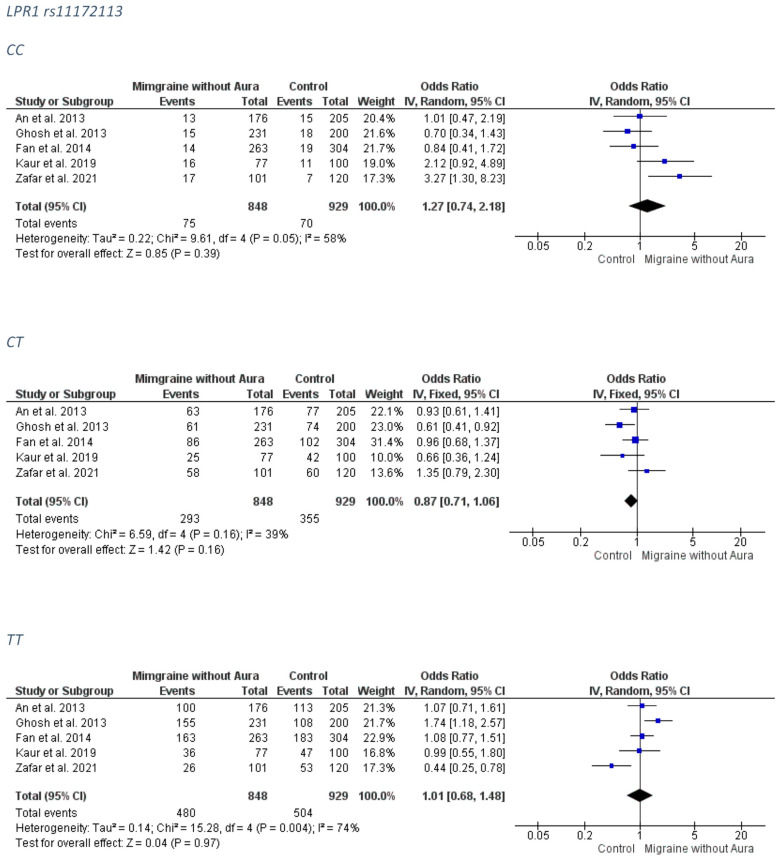
The forest plots presenting the results from meta-analysis of the rs11172113 and migraine without aura group.

No association was detected between the LPR1 rs11172113 and MO subgroup. The forest plots can be accessed in Figure 9. Analysis for publication bias (Supplementary File S14) suggested that smaller, less precise studies tended to overestimate the true effect (OR) of the CC model, which was probably closer to 1.00 than estimated (i.e., no association), and mildly underestimated the true effect of the recessive model TT. The CT model appears to be less (if at all) affected by publication bias.

## 4. Discussion

In this meta-analysis, we investigated the effect of three variants (namely the PRDM16 rs2651899, the rs10166942 near the TRPM8 gene, and the LPR1 rs11172113) on the risk of migraine, as well as on the risk of the main migraine phenotypes, namely the MA and the MO. Our study detected a significant influence of the PRDM16 rs2651899 on the risk of overall migraine and MA. Moreover, we detected a significant association between the rs10166942 (near the TRPM8 gene) CT genotype and increased migraine risk and MO risk, while the homozygosities appear to confer a protective effect. Finally, we did not detect any association between the LRP1 rs11172113 and any of the migraine phenotypes.

The PRDM16 gene encodes a zinc finger transcription factor, which contains an N-terminal PR domain [59,60]. The precise mechanism by which PRDM16 may be involved in migraine remains unknown. There are indications that the PRDM16 may be implicated in a molecular mechanism related to brown extra fat cells and preadipocytes, and as such, it may possibly be related to obesity [61]. This is of great interest, considering that obesity (total body and abdominal) has been associated with an increased prevalence of migraine and frequency of migraine attacks [62]. Moreover, the PRDM16 is implicated in oxidative stress and neurogenesis [63]. Such mechanisms have also been implicated in migraine pathogenesis [64,65,66].

The PRDM16 rs2651899 polymorphism is an intronic variant located at chromosome 1:3167148. This variant polymorphism may alter PRDM16 gene splicing or may have an effect on downstream regulatory elements, influencing the expression of PRDM16 mRNA [37]. The variety in the direction of the association between migraine and the rs2651899 (meaning that both the alleles have been reported to be associated with the migraine risk), denotes that the possible biological consequences of the rs2651899 on the PRDM16 polymorphism are far from being fully elucidated. This could possibly explain the fact that we did not detect any association between the PRDM16 rs2651899 and MO, as in a previous meta-analysis [37].

TRPM8 proteins are cold-sensitive channels responding to a great variety of ligands [67]. They are primarily expressed on peripheral sensory neurons and also on sensory afferents of the meninges [68,69]. Exposure to cold temperatures is a known trigger of migraine attacks [70]. While it is not clear whether meningeal TRPM8 protein are sensitive to weather fluctuations [70], it has been observed that activation of meningeal TRPM8 can alter the feeling of pain [67]. TRPM8 has also drawn attention as it is considered as a possible therapeutic target for migraine, neuropathic pain, and non-headache disorders [67,71,72,73].

The rs10166942 is an upstream gene variant located at Chromosome 2:233916448, near the TRPM8 gene. Interestingly, carriers of the rs10166942 C allele appeared to have decreased TRPM8 expression and reduced sensitivity to cold stimuli [74]. Moreover, carriers of the rs10166942 T allele presented more allodynic symptoms compared with the non-T allele carriers [53].

The third examined polymorphism in our study is the intronic rs11172113 located at Chromosome 12:57133500 of the LRP1 gene. The LRP1 gene is expressed in the brain, vasculature and, many other human tissues [75]. It is implicated in synaptic transmission, neuronal calcium signaling, amyloid precursor protein metabolism, and neuronal and glutamate signaling. [75]. Considering that elevated interictal glutamate levels have been found in the visual cortex of patients with MA, cortical hyperexcitability may be among the pathophysiological mechanisms that connect LRP1 with migraine [76,77,78]. Notably, our study did not detect any association between migraine and LRP1 rs11172113.

The fact that none of the examined polymorphisms has been associated with both MA and MO subgroups could be attributed to several reasons. Firstly, despite the similarities in genetic architecture between MA and MO, a few differences also exist [23]. Moreover, patients with MA were fewer than participants with MO, and obviously both the MA and MO datasets were smaller compared to the overall migraine group, suggesting that the analysis of the subgroup may lack the statistical power needed to detect a possible association with the tested variants.

Migraine causes severe impairment, influencing the quality of life, and the patients are unable to be productive in their daily activities [79,80]; consequently, migraine has a considerable economic impact on societies [81]. In an attempt to offer effective and personalized treatment, genetic studies are paving the way towards “precision medicine” targeted healthcare strategies, that take into account an individual’s genetic make-up and other environmental factors, to offer the optimal therapeutic and preventive options for each case. However, whether or not the variants meta-analyzed in our study could eventually have an impact on the diagnosis, prognosis, and treatment response remains elusive, highlighting the necessity for research on migraine, given the high prevalence in patients who suffer from this disease [14,82,83].

## 5. Conclusions

In conclusion, based on our findings the PRDM16 rs2651899 is associated with migraine and MA, and the rs10166942 (near the TRPM8 gene) CT genotype is associated with increased migraine risk and MO risk, while the homozygosities appear to confer a protective effect. Additionally, we did not detect any association between the LRP1 rs11172113 and any of the migraine phenotypes. In any case, considering that therapeutic approaches for migraine are often ineffective, it will be interesting to observe whether a personalized treatment based on the genetic architecture of each individual could be applied in the future. Future larger collaborative studies are needed, in cohorts with multiethnic backgrounds, for the role of these variants in migraine to be more accurately elucidated.

## Data Availability

Not applicable.

## Supplementary Materials

The following supporting information can be downloaded at: https://www.mdpi.com/article/10.3390/medicina58040491/s1, Supplementary File S1. PRISMA 2009 checklist; Supplementary File S2. The PubMed search algorithm; Supplementary File S3. The flowchart presenting the selection procedure of eligible studies for the rs2651899; Supplementary File S4. The flowchart presenting the selection procedure of eligible studies for the rs10166942; Supplementary File S5. The flowchart presenting the selection procedure of eligible studies for the rs11172113; Supplementary File S6. The funnel plots presenting the results from meta-analysis of the rs2651899 and overall migraine group; Supplementary File S7. The funnel plots presenting the results from meta-analysis of the rs2651899 and migraine with aura group; Supplementary File S8. The funnel plots presenting the results from meta-analysis of the rs2651899 and migraine without aura group; Supplementary File S9. The funnel plots presenting the results from meta-analysis of the rs10166942 and overall migraine group; Supplementary File S10.The funnel plots presenting the results from meta-analysis of the rs10166942 and migraine with aura group; Supplementary File S11. The funnel plots presenting the results from meta-analysis of the rs10166942 and migraine without aura group; Supplementary File S12. The funnel plots presenting the results from meta-analysis of the rs11172113 and overall migraine group; Supplementary File S13. The funnel plots presenting the results from meta-analysis of the rs11172113 and migraine with aura group; Supplementary File S14. The funnel plots presenting the results from meta-analysis of the rs11172113 and migraine without aura group.
