# Supplementary material for: Deciphering the Role of the rs2651899, rs10166942, and rs11172113 Polymorphisms in Migraine: A Meta-Analysis
Source: Medicina (Kaunas). 2022 Mar 29;58(4):491. doi: 10.3390/medicina58040491 (PMC9031971; doi:10.3390/medicina58040491)
Supplement: Supplementary file 1 [file medicina-58-00491-s001.zip › Supplementary File S2.pdf]

rs2651899

"(""migrain""[All Fields] OR ""migraine disorders""[MeSH Terms] OR (""migraine""[All Fields] AND ""disorders""[All Fields]) OR ""migraine disorders""[All Fields] OR ""migraine""[All Fields] OR ""migraines""[All Fields] OR ""migraine s""[All Fields] OR ""migraineous""[All Fields] OR ""migrainers""[All Fields] OR ""migrainous""[All Fields]) AND ""rs2651899""[All Fields]"

rs10166942

"(""migrain""[All Fields] OR ""migraine disorders""[MeSH Terms] OR (""migraine""[All Fields] AND ""disorders""[All Fields]) OR ""migraine disorders""[All Fields] OR ""migraine""[All Fields] OR ""migraines""[All Fields] OR ""migraine s""[All Fields] OR ""migraineous""[All Fields] OR ""migrainers""[All Fields] OR ""migrainous""[All Fields]) AND ""rs10166942""[All Fields]"

rs11172113

" (""migrain""[All Fields] OR ""migraine disorders""[MeSH Terms] OR (""migraine""[All Fields] AND ""disorders""[All Fields]) OR ""migraine disorders""[All Fields] OR ""migraine""[All Fields] OR ""migraines""[All Fields] OR ""migraine s""[All Fields] OR ""migraineous""[All Fields] OR ""migrainers""[All Fields] OR ""migrainous""[All Fields]) AND ""rs11172113""[All Fields]"
