# Supplementary material for: Deciphering the Role of the rs2651899, rs10166942, and rs11172113 Polymorphisms in Migraine: A Meta-Analysis
Source: Medicina (Kaunas). 2022 Mar 29;58(4):491. doi: 10.3390/medicina58040491 (PMC9031971; doi:10.3390/medicina58040491)
Supplement: Supplementary file 1 [file medicina-58-00491-s001.zip › Supplementary File S5.pdf]

PRISMA 2009 Flow Diagram

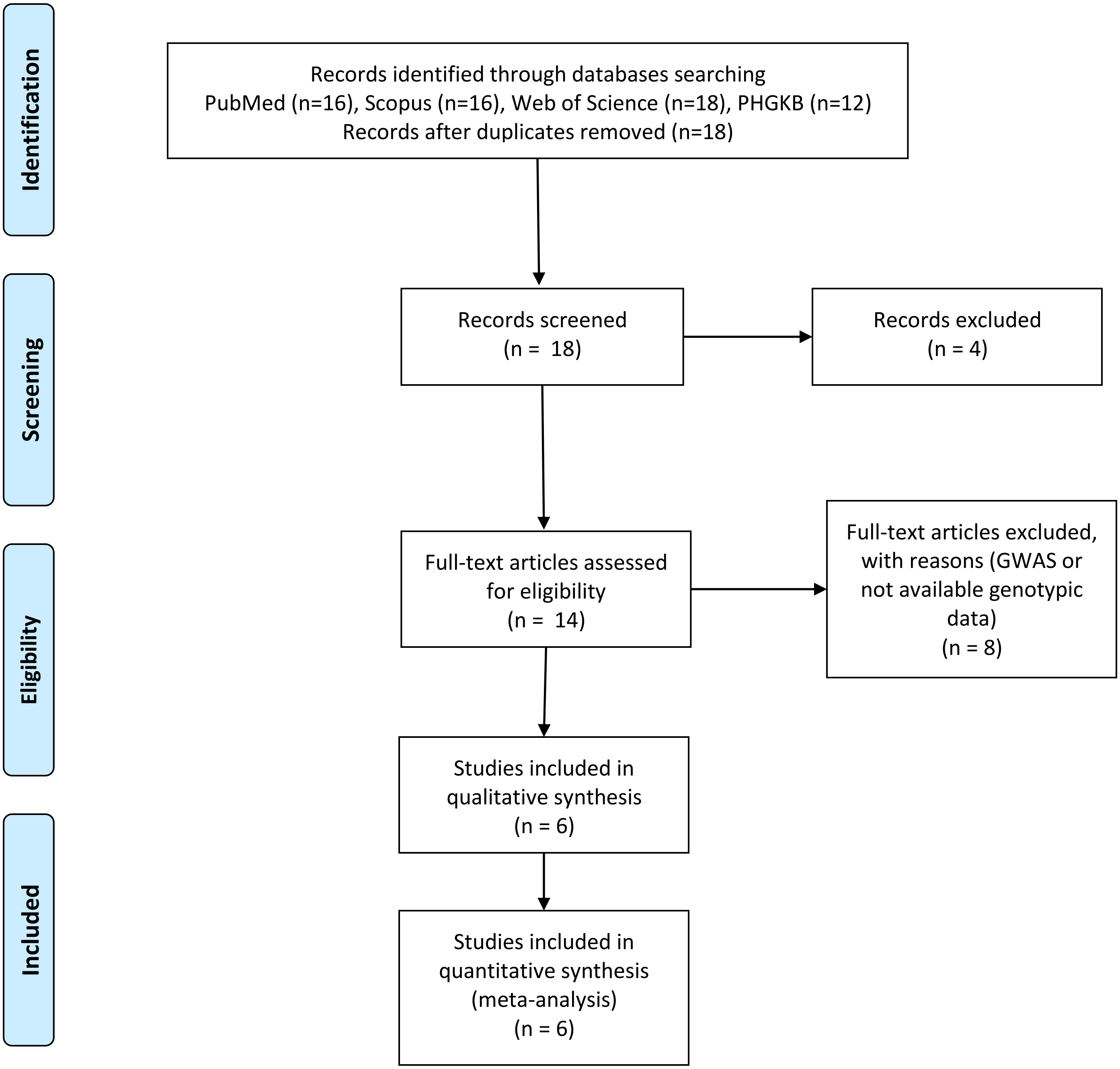

From: Moher D, Liberati A, Tetzlaff J, Altman DG, The PRISMA Group (2009). *P*referred *R*eporting *I*tems for *S*ystematic Reviews and *M*eta-*A*nalyses: The PRISMA Statement. PLoS Med 6(6): e1000097. doi:10.1371/journal.pmed1000097

For more information, visit www.prisma-statement.org.
