# Supplementary figures and images for: Deciphering the Role of the rs2651899, rs10166942, and rs11172113 Polymorphisms in Migraine: A Meta-Analysis
Source: Medicina (Kaunas). 2022 Mar 29;58(4):491. doi: 10.3390/medicina58040491 (PMC9031971; doi:10.3390/medicina58040491)

rs10166942 near TRPM8 gene

CC

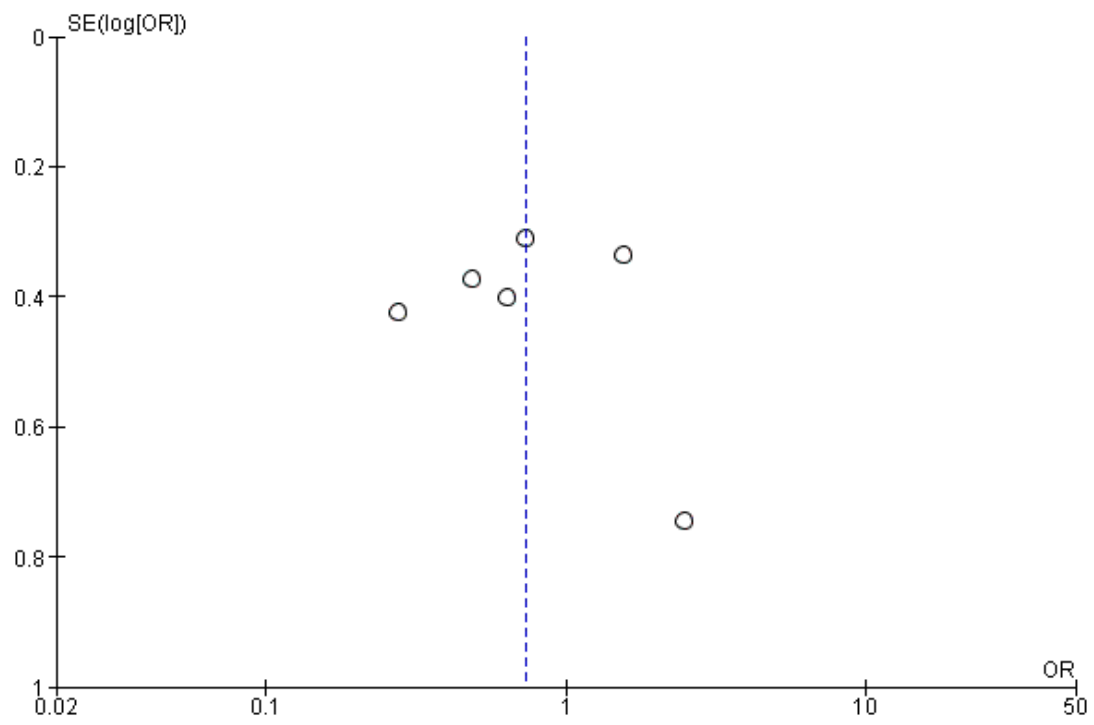

CT

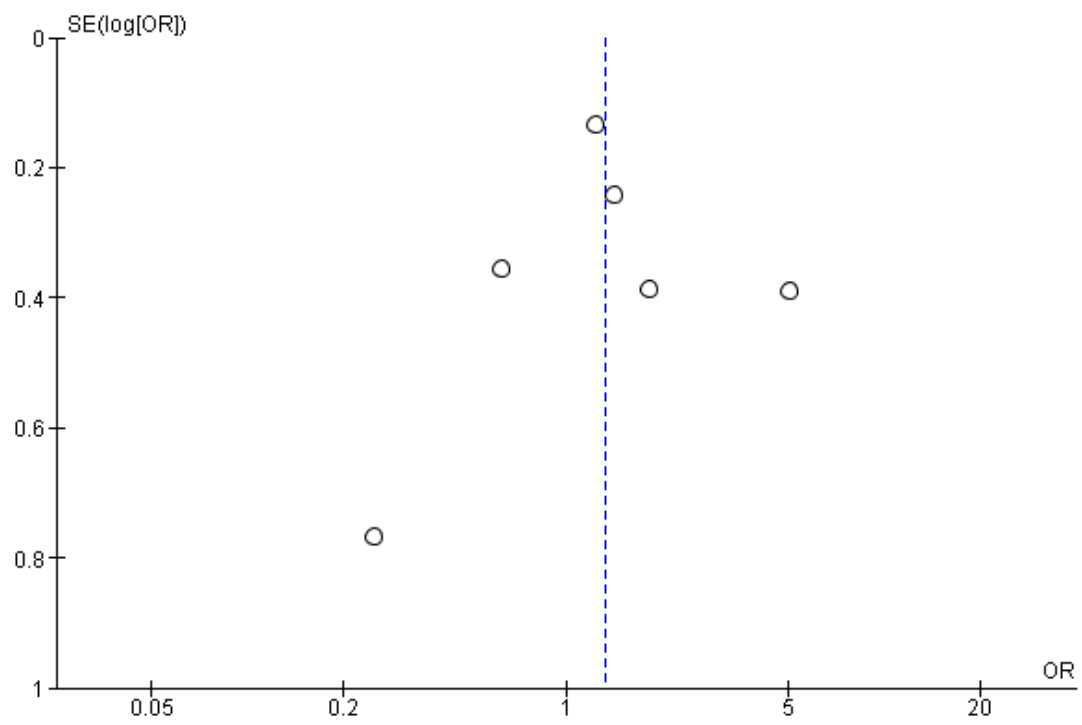

$\pi$

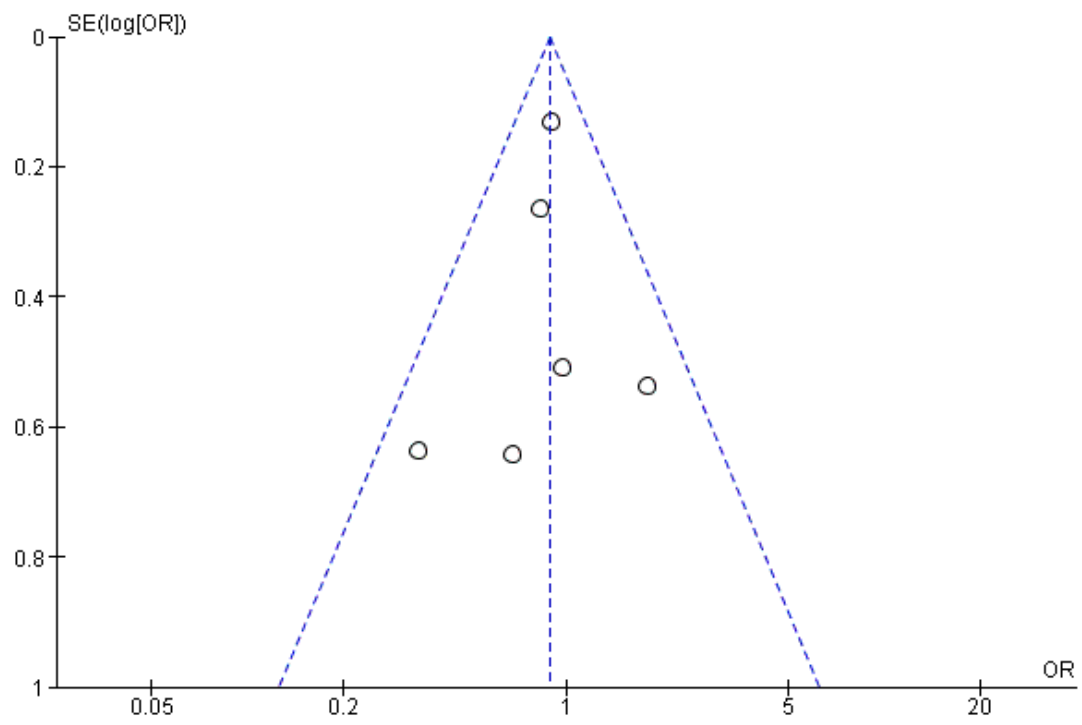

Supplement: Supplementary file 1 [file medicina-58-00491-s001.zip › Supplementary File S10.pdf]

rs10166942 near TRPM8 gene

CC

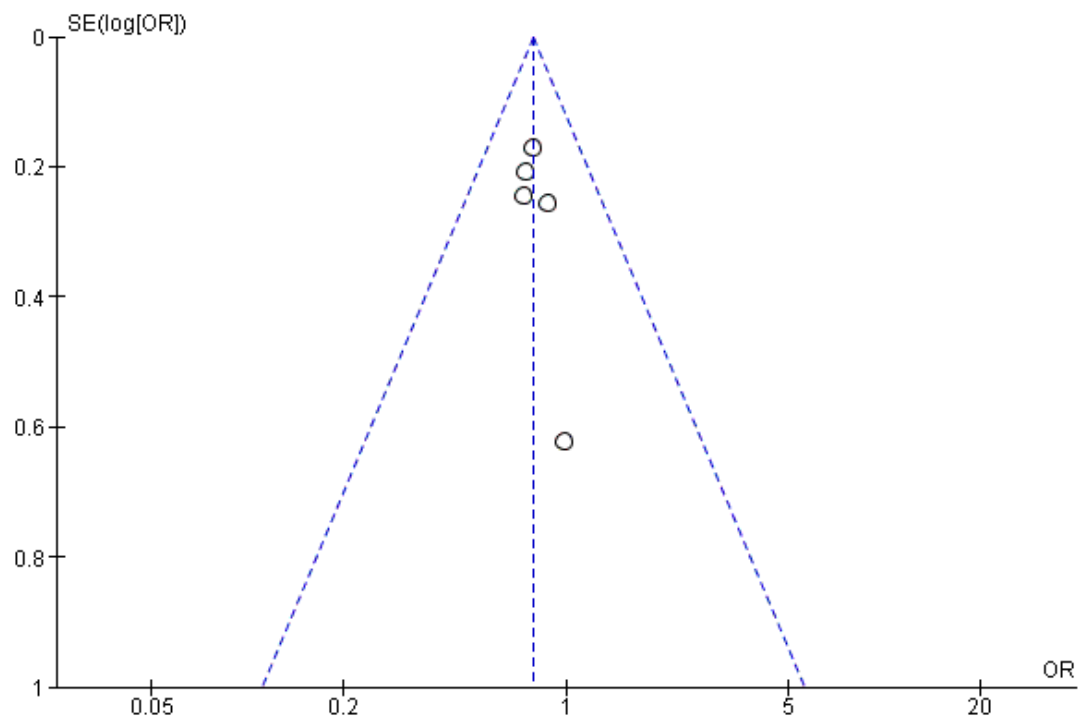

CT

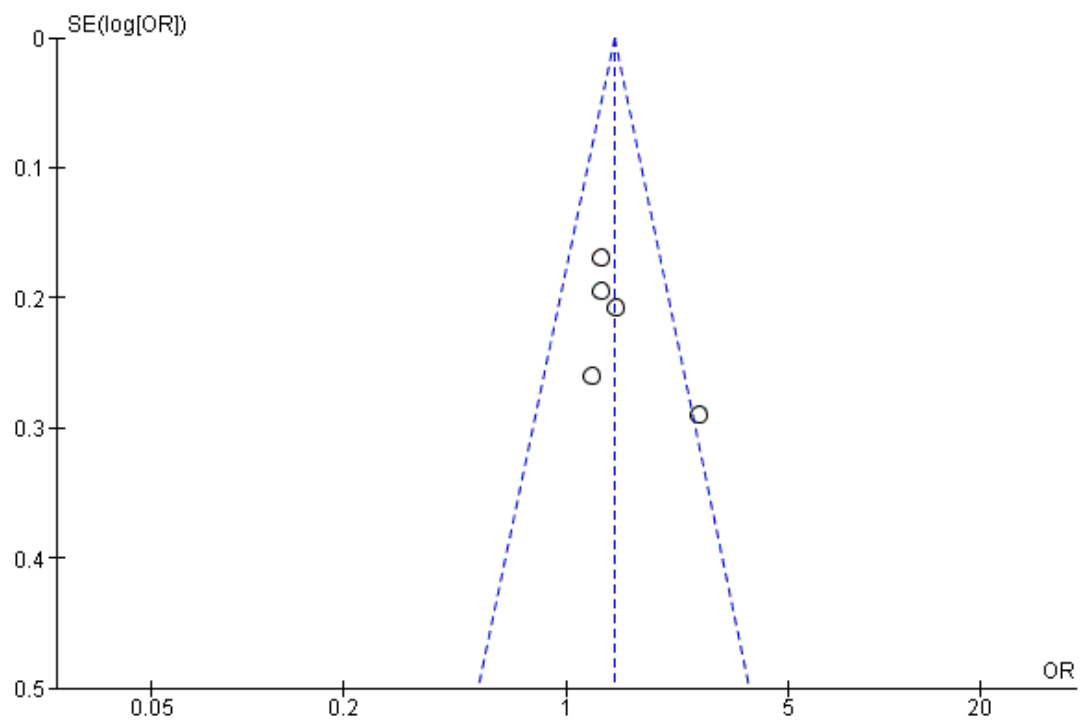

$\pi$

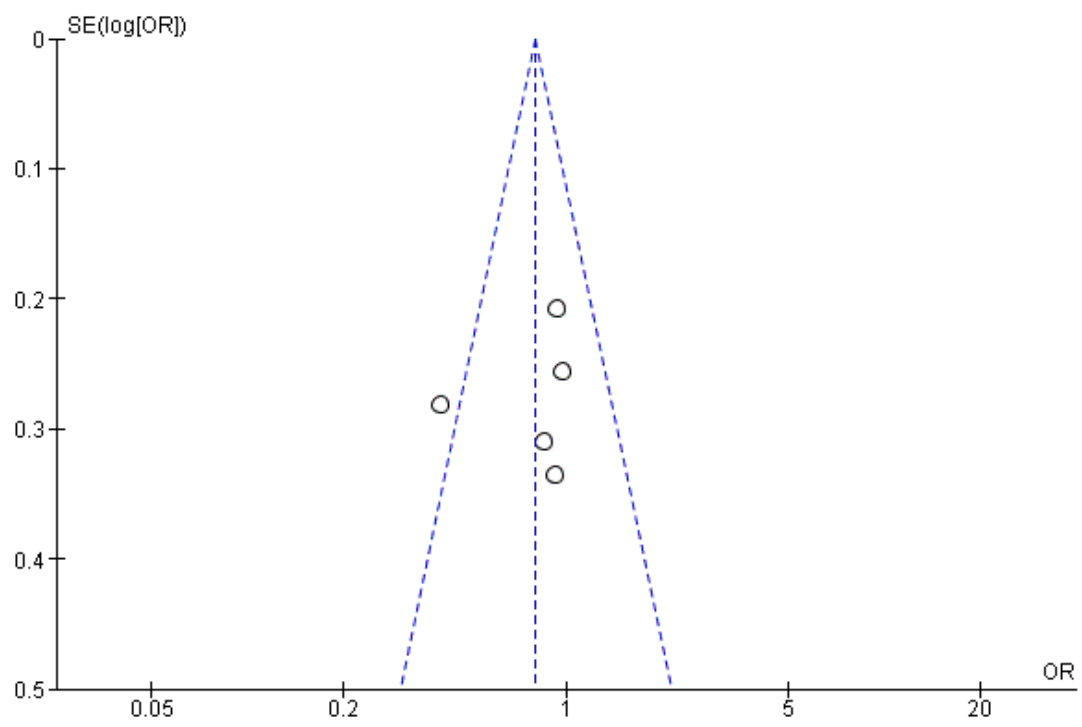

Supplement: Supplementary file 1 [file medicina-58-00491-s001.zip › Supplementary File S11.pdf]

*LPR1 rs11172113*

*CC*

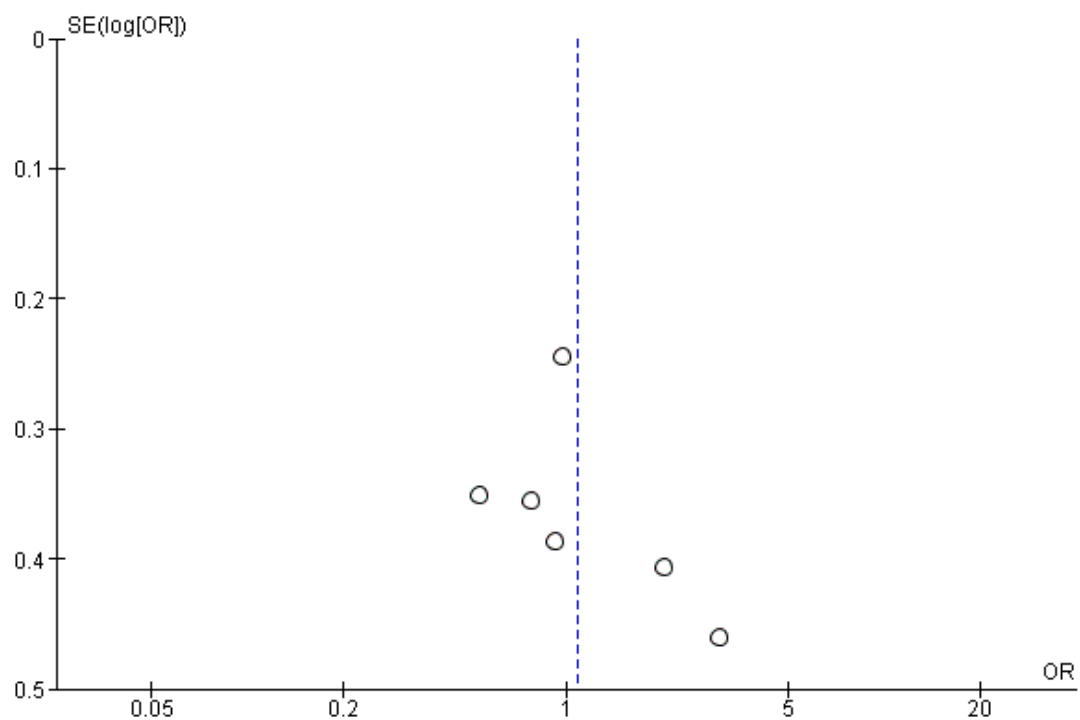

*CT*

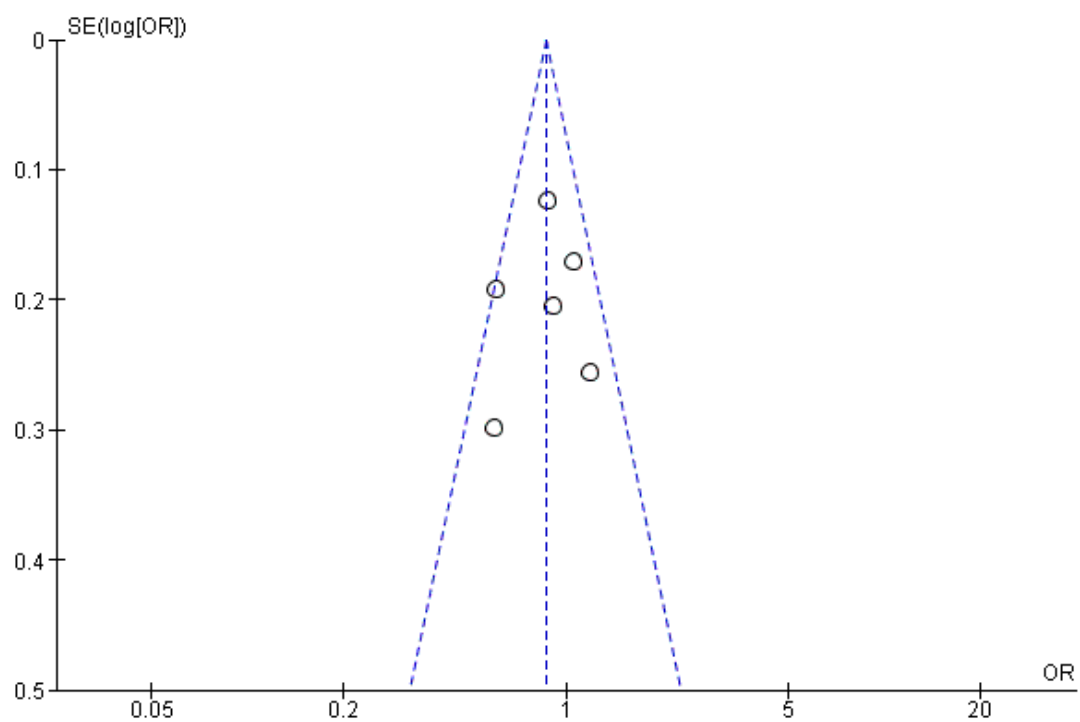

$\pi$

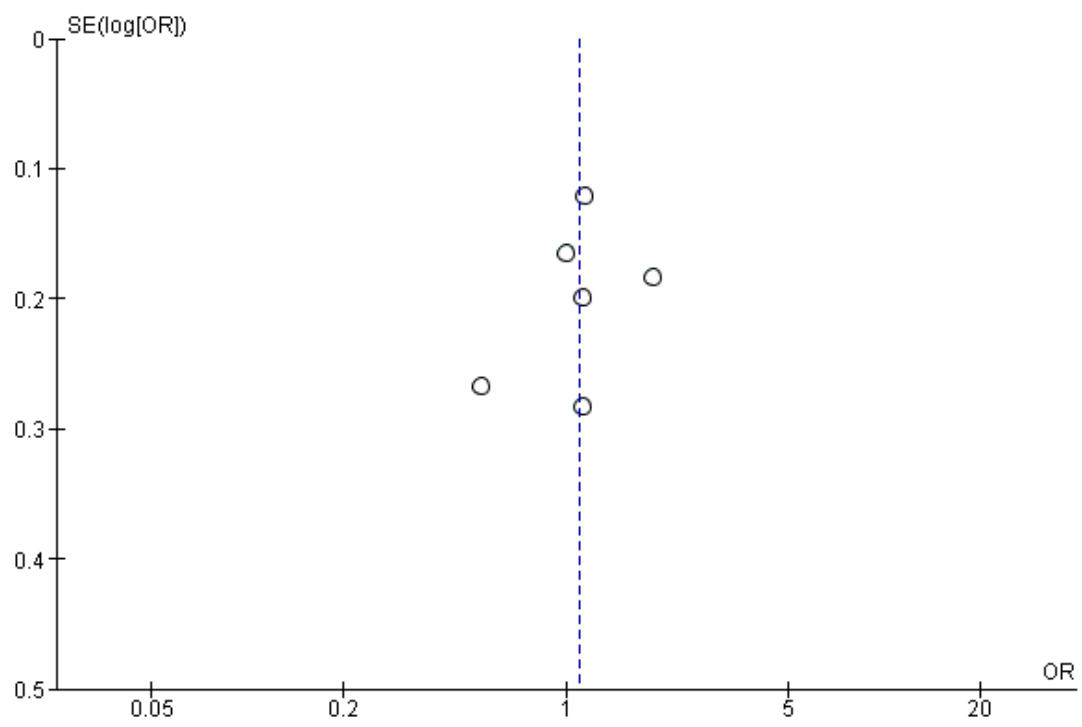

Supplement: Supplementary file 1 [file medicina-58-00491-s001.zip › Supplementary File S12.pdf]

*LPR1 rs11172113*

*CC*

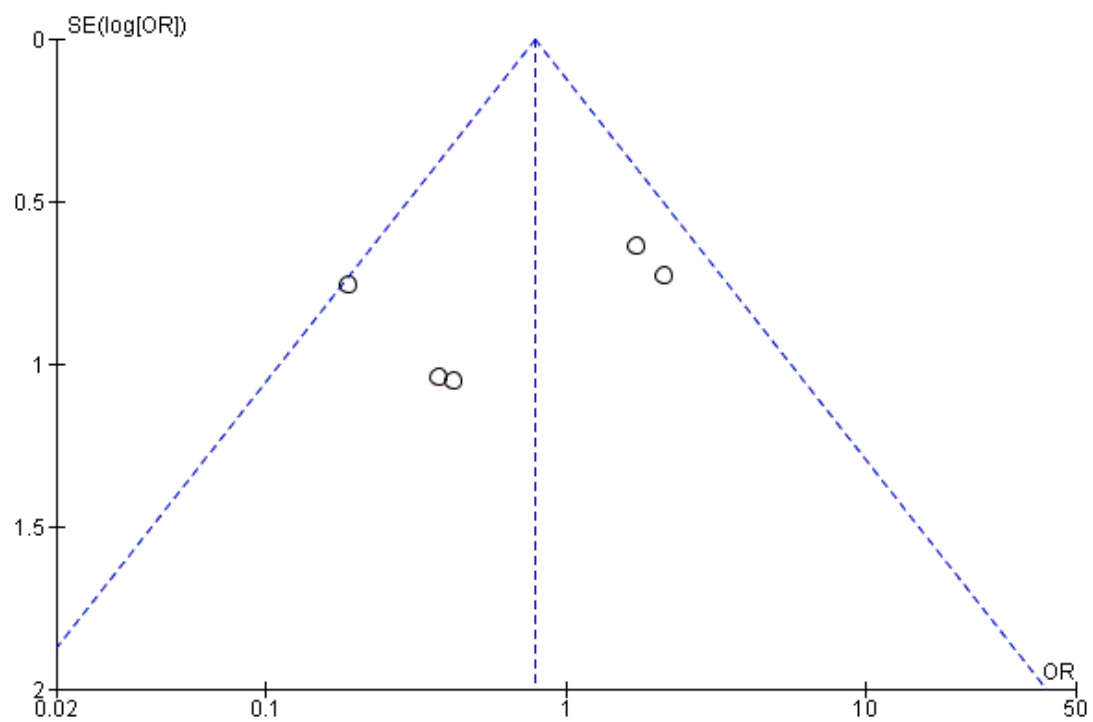

*CT*

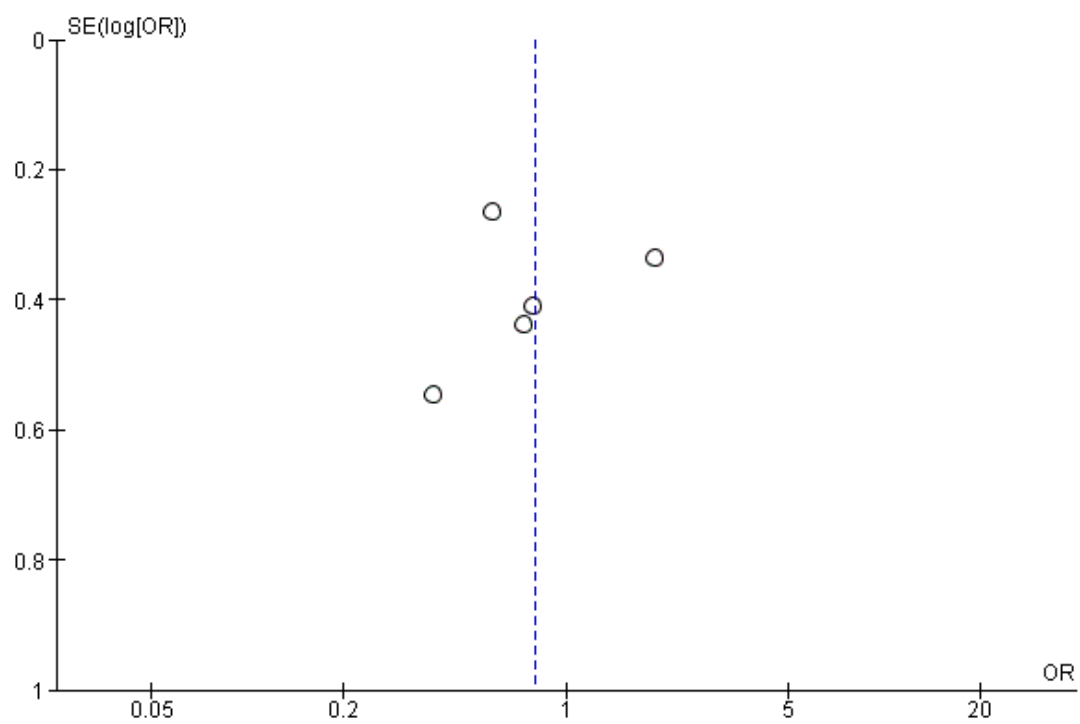

$\pi$

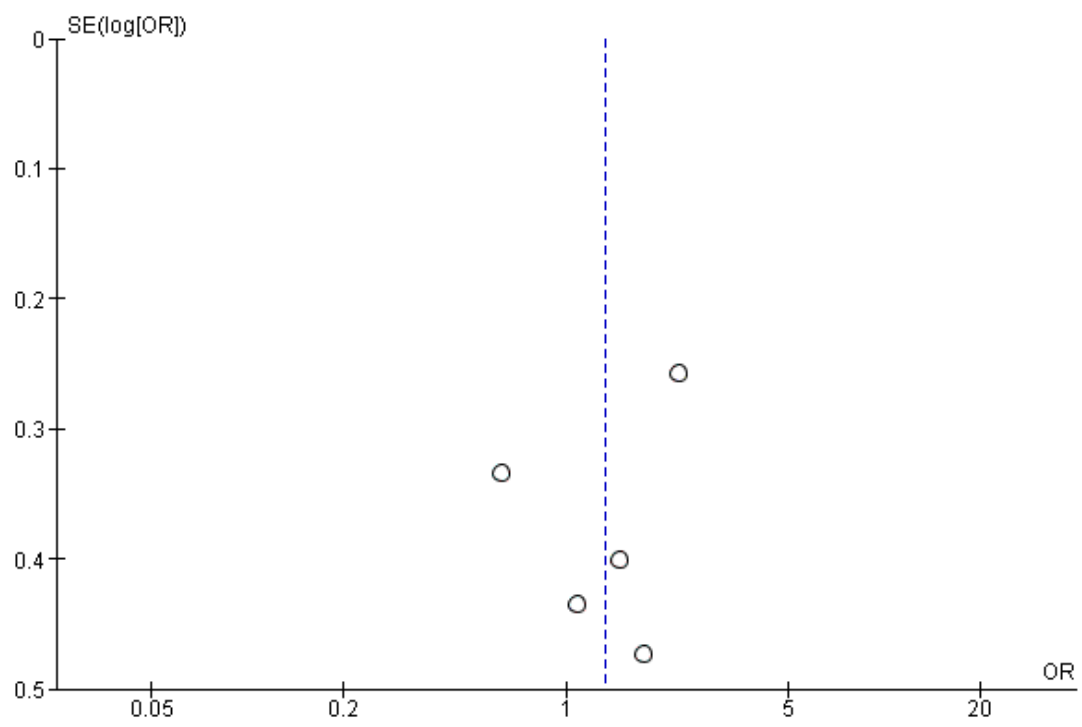

Supplement: Supplementary file 1 [file medicina-58-00491-s001.zip › Supplementary File S13.pdf]

*LPR1 rs11172113*

*CC*

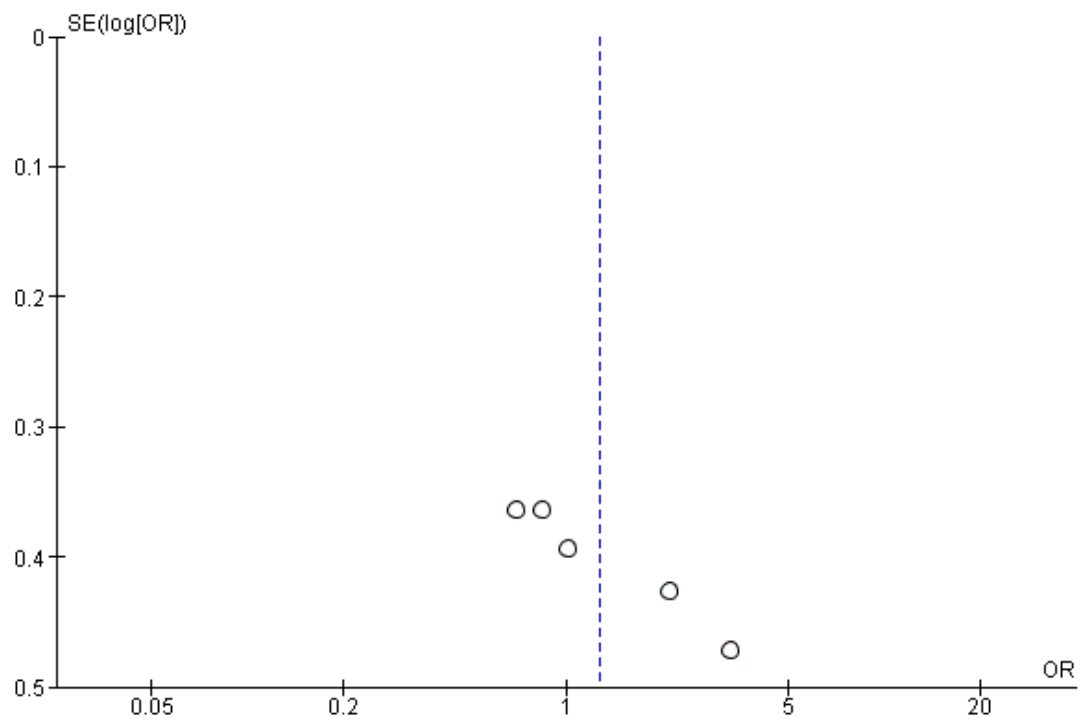

*CT*

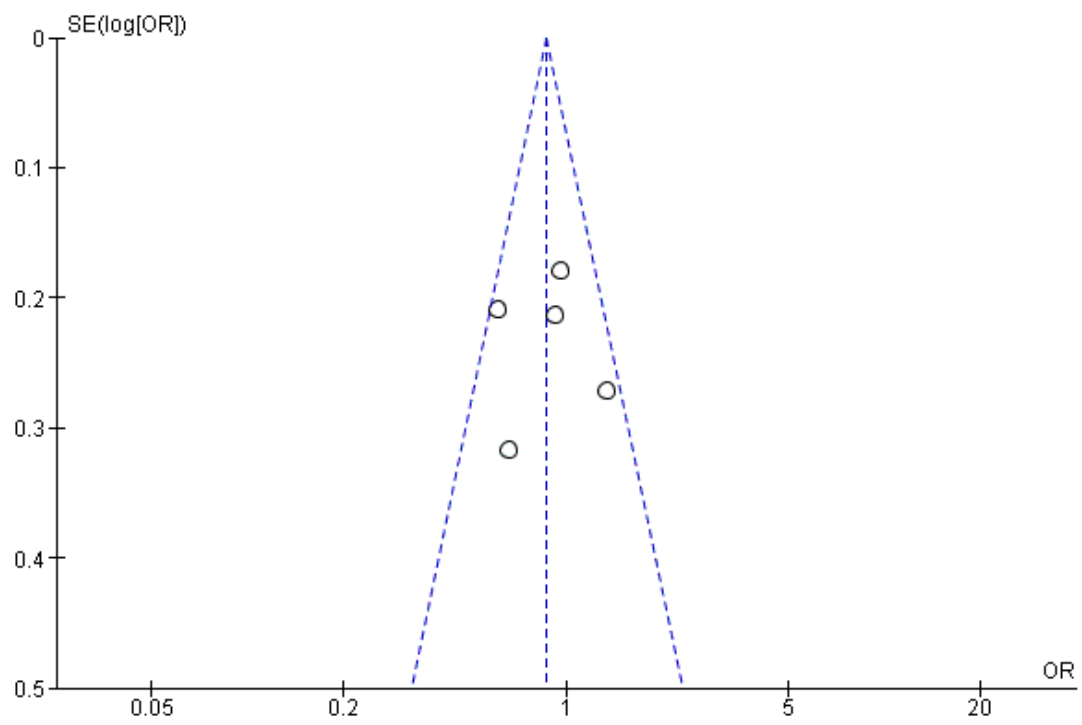

$\pi$

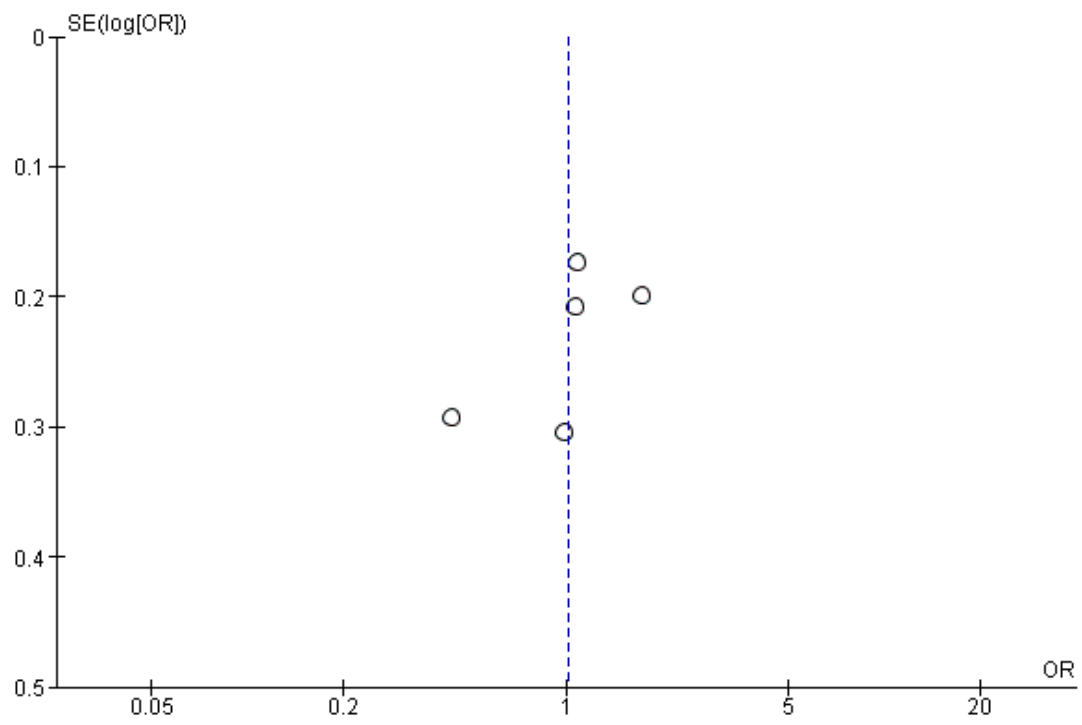

Supplement: Supplementary file 1 [file medicina-58-00491-s001.zip › Supplementary File S14.pdf]

PRDM16 rs2651899

CC

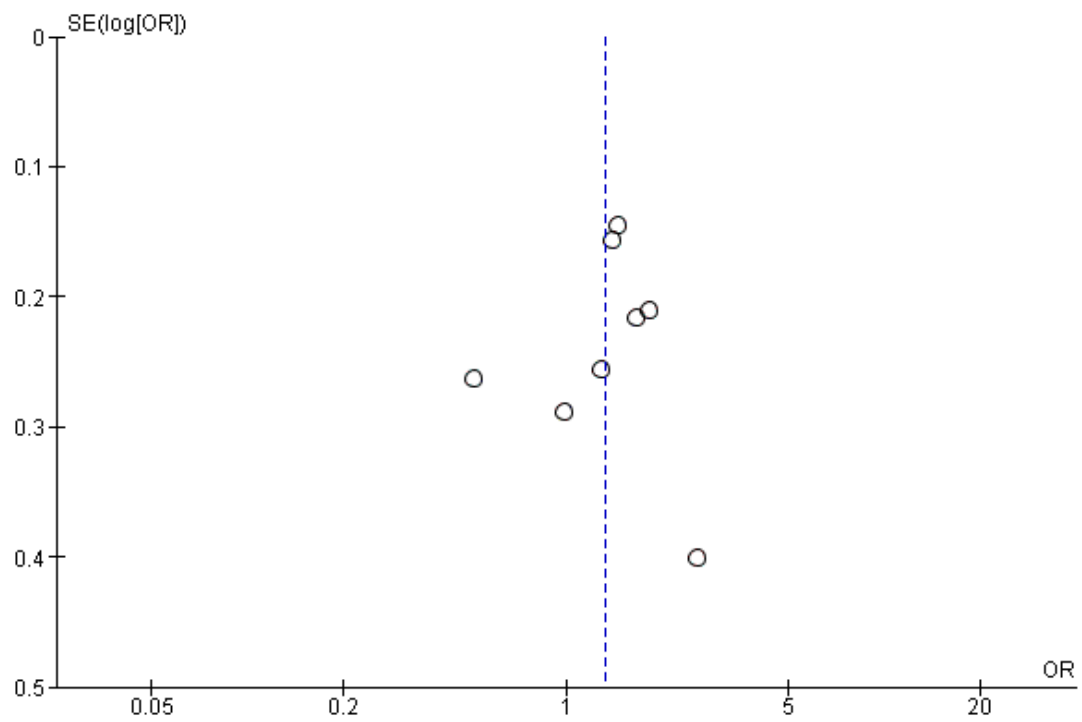

CT

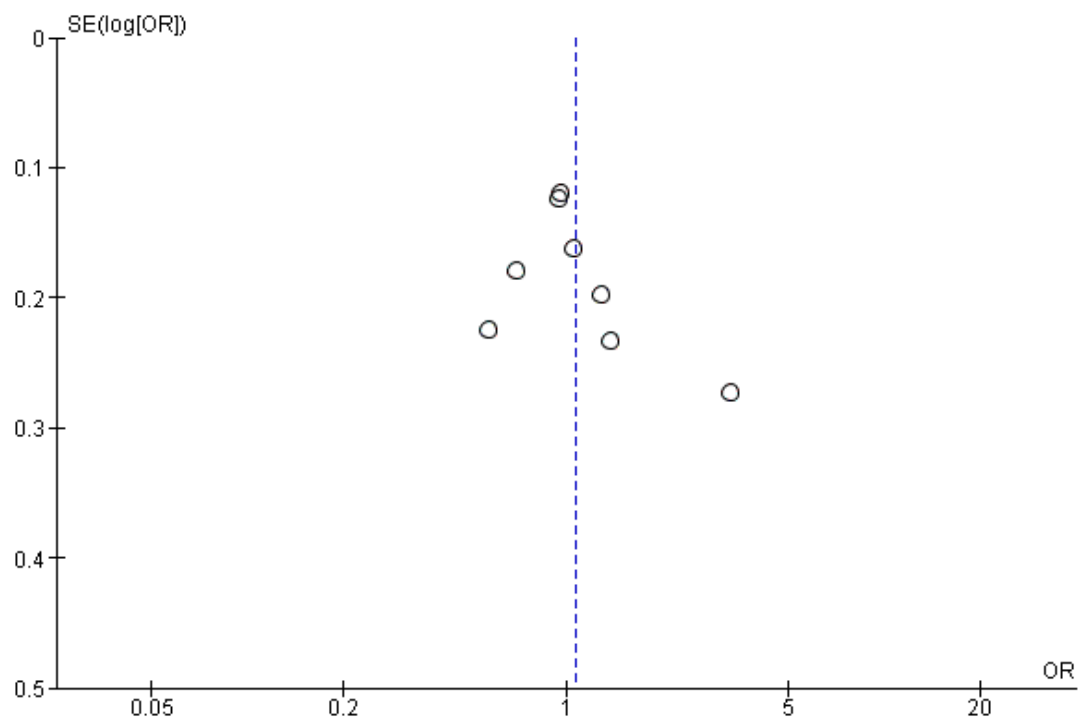

$\pi$

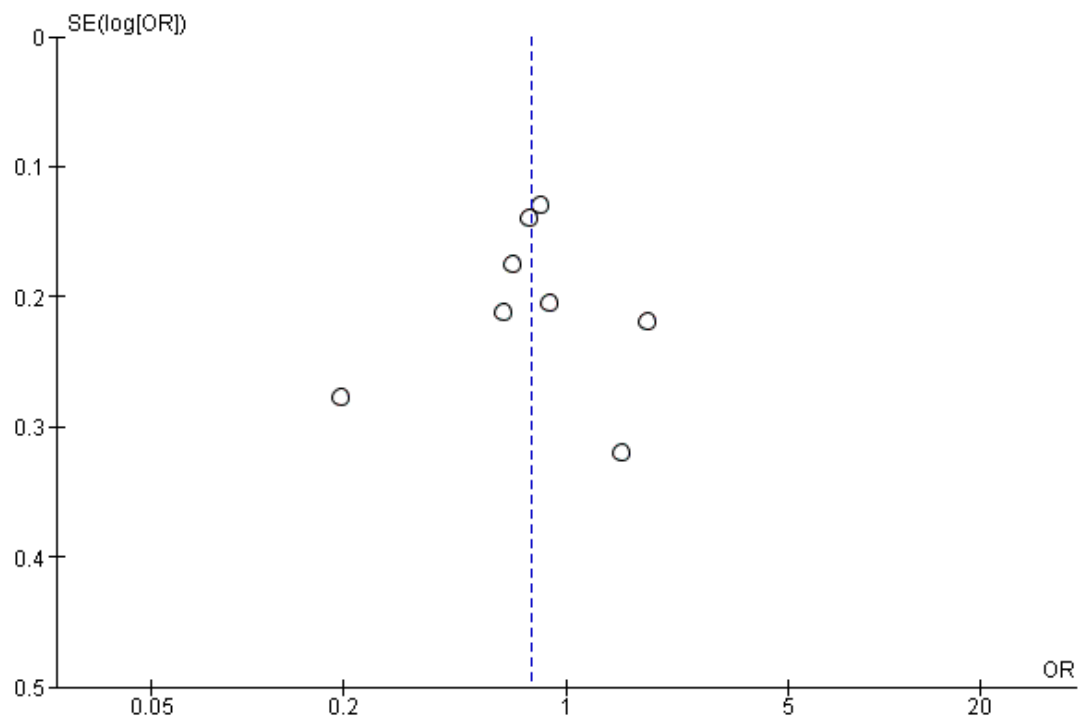

Supplement: Supplementary file 1 [file medicina-58-00491-s001.zip › Supplementary File S6.pdf]

CC

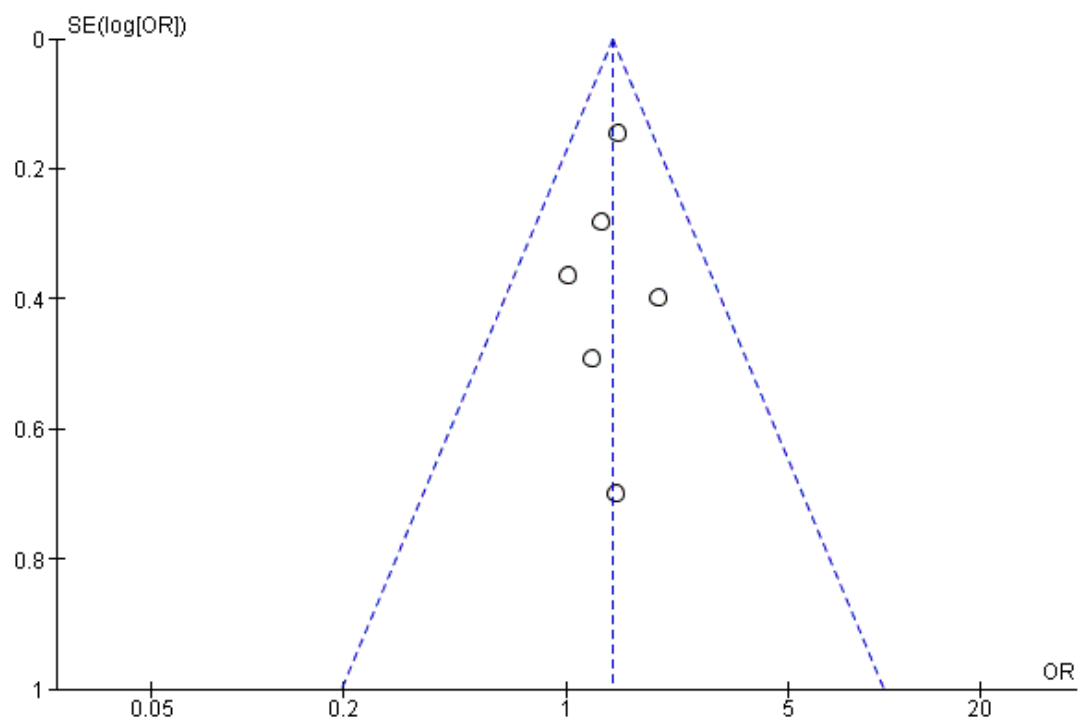

CT

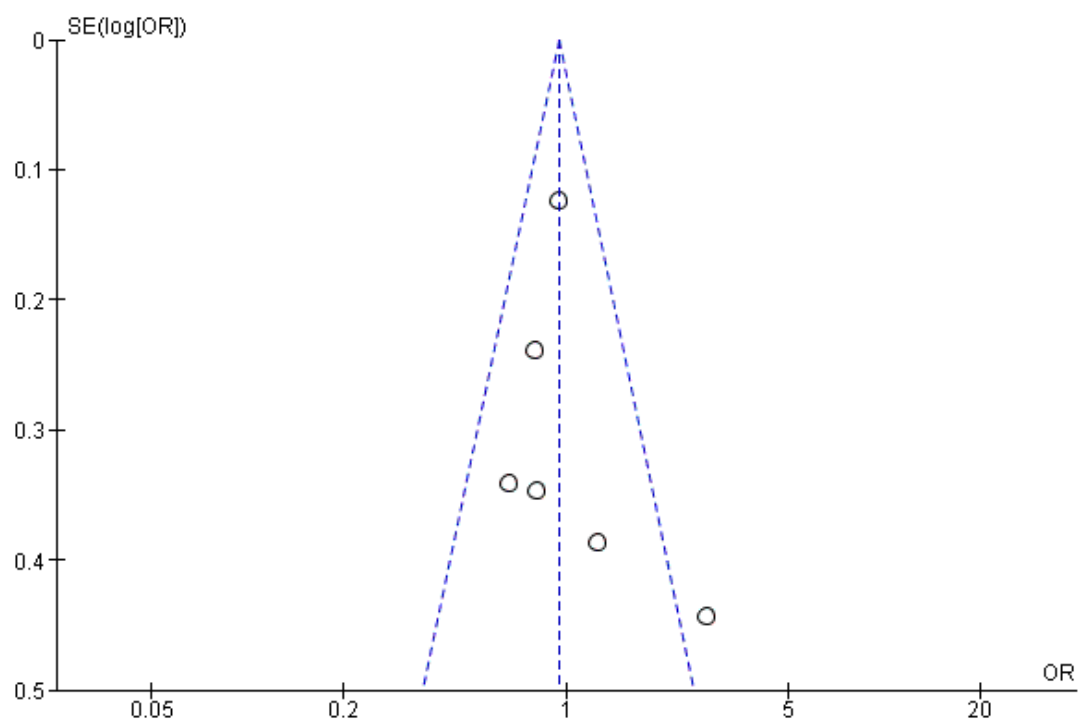

$IT$

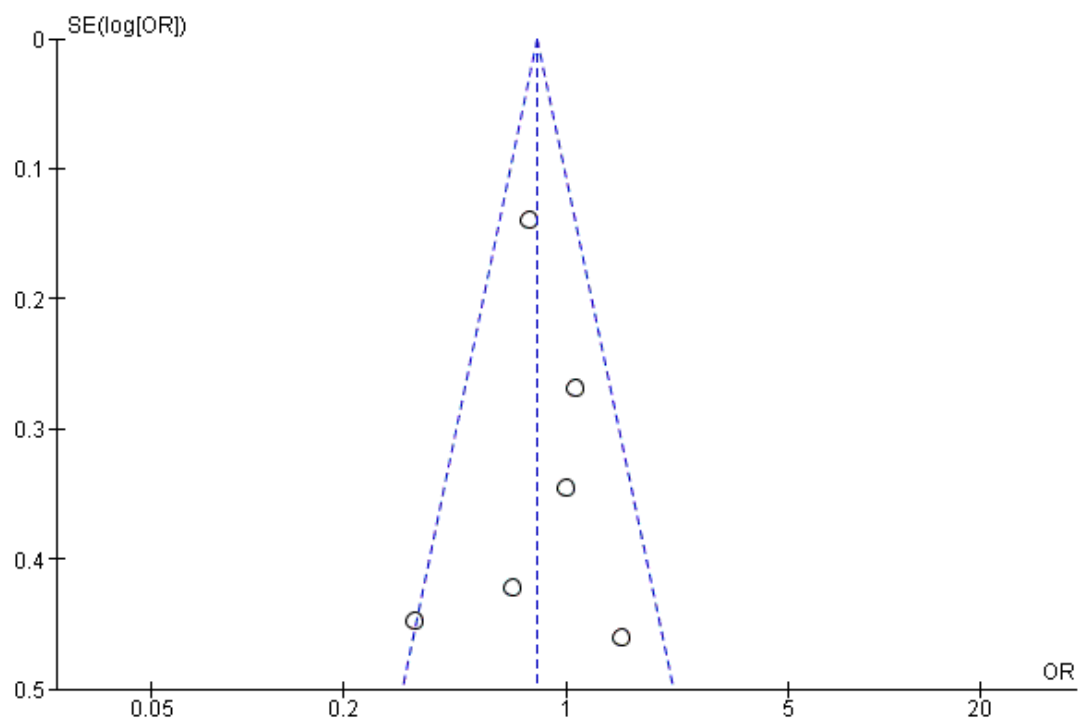

Supplement: Supplementary file 1 [file medicina-58-00491-s001.zip › Supplementary File S7.pdf]

PRDM16 rs2651899

CC

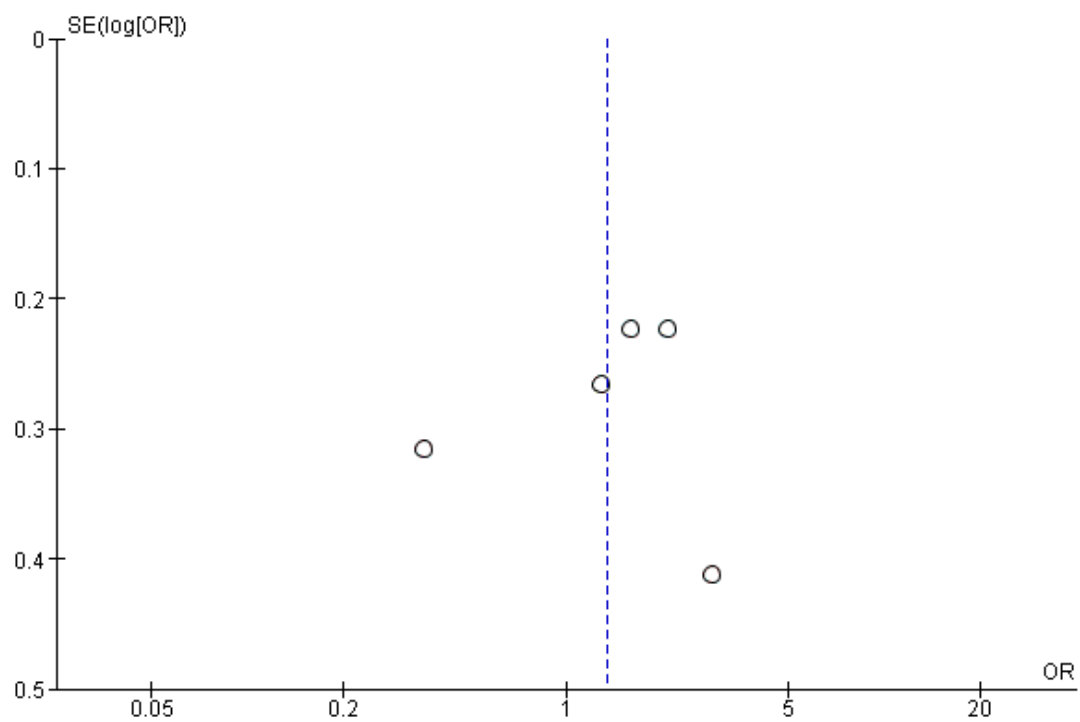

CT

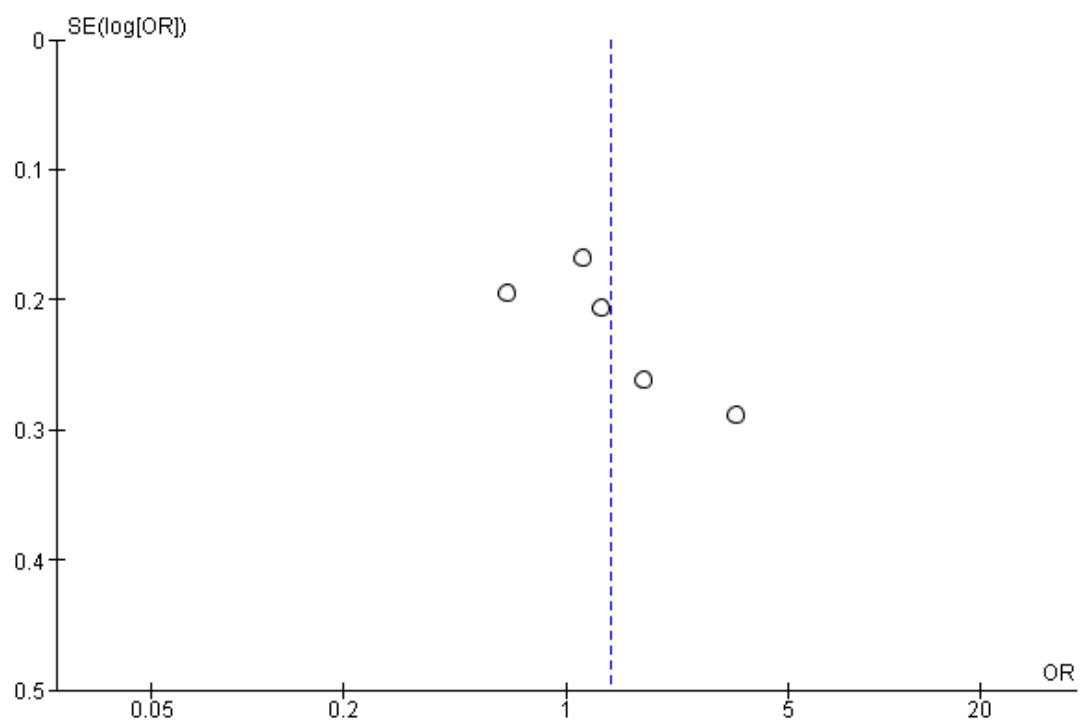

$\pi$

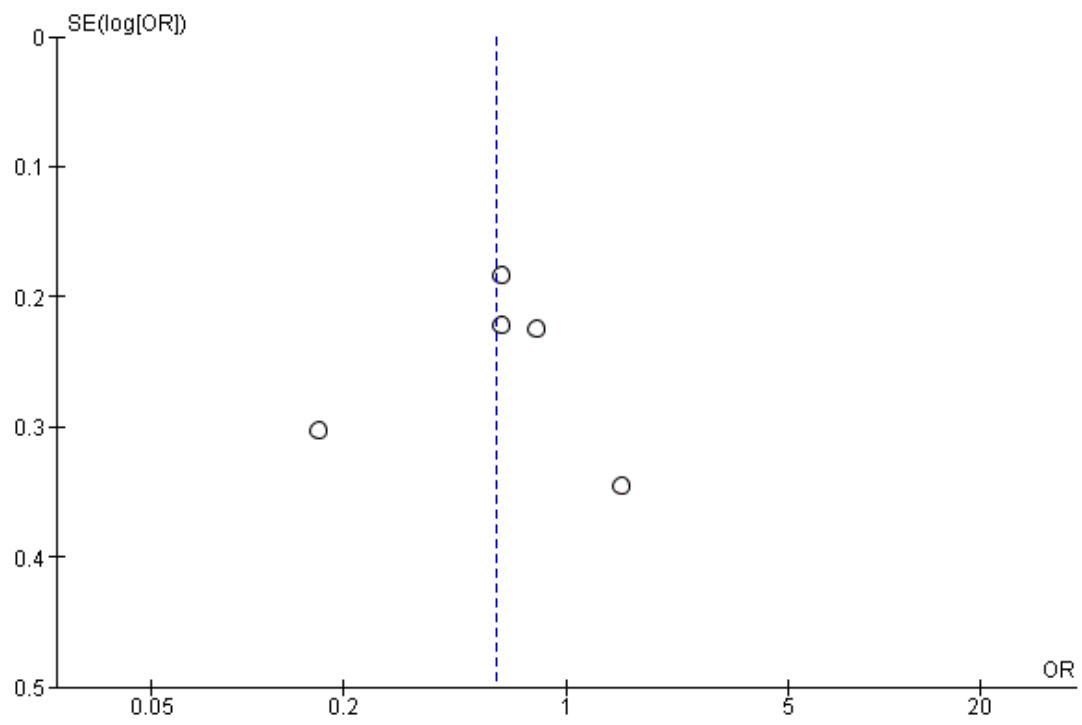

Supplement: Supplementary file 1 [file medicina-58-00491-s001.zip › Supplementary File S8.pdf]

rs10166942 near TRPM8 gene

CC

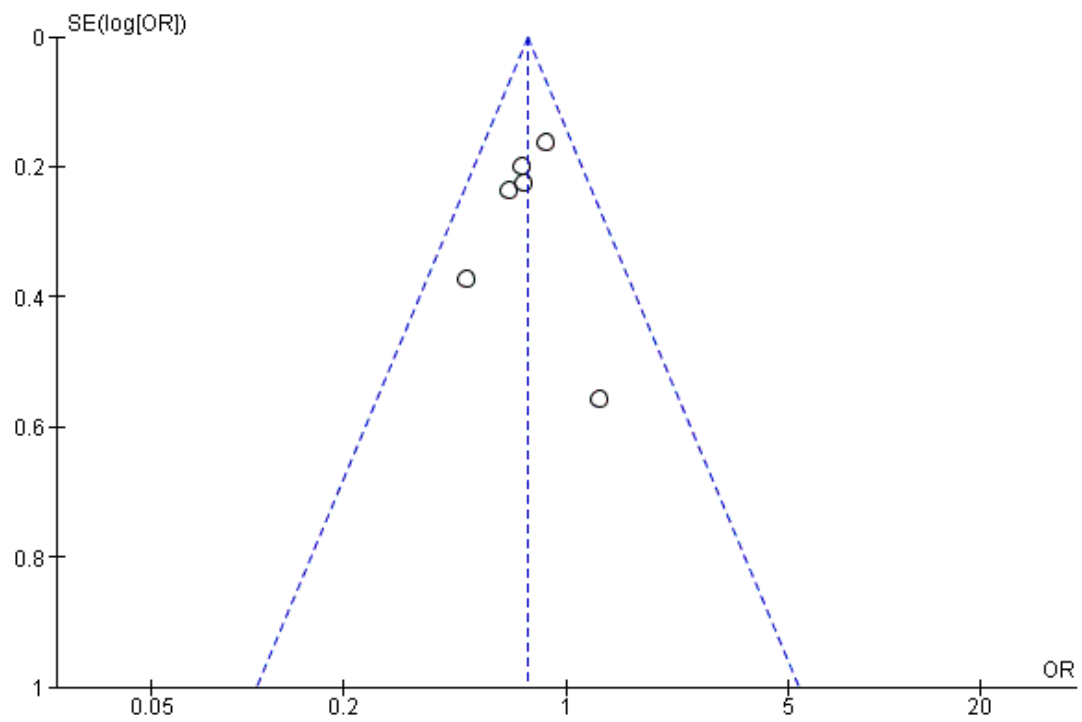

CT

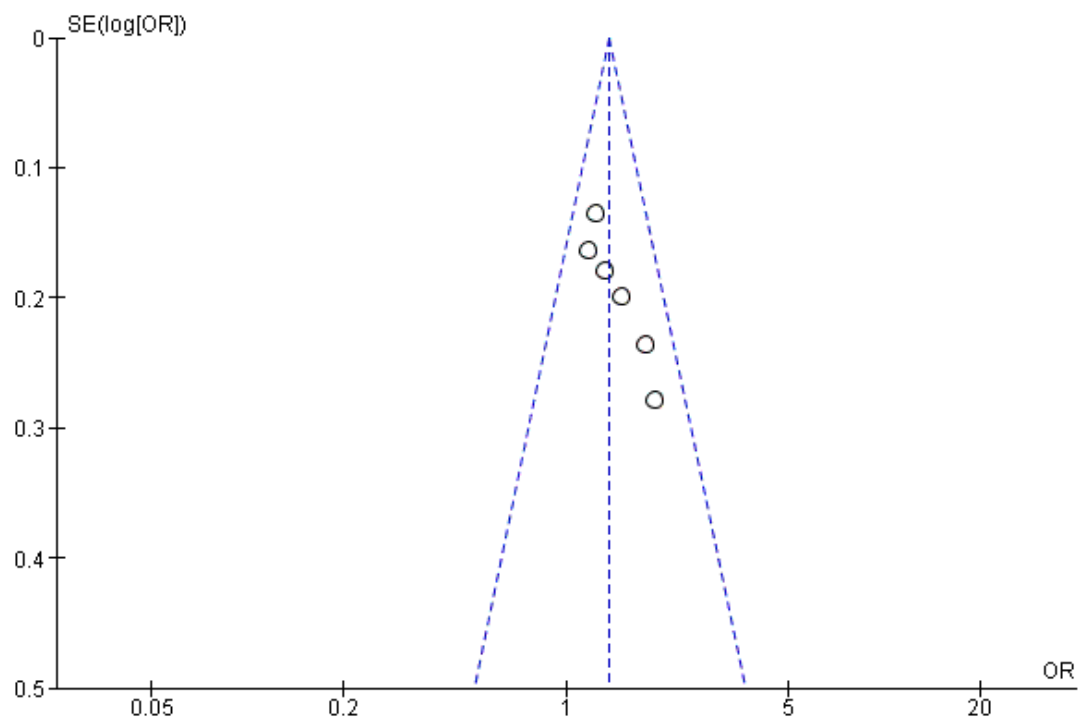

$\pi$

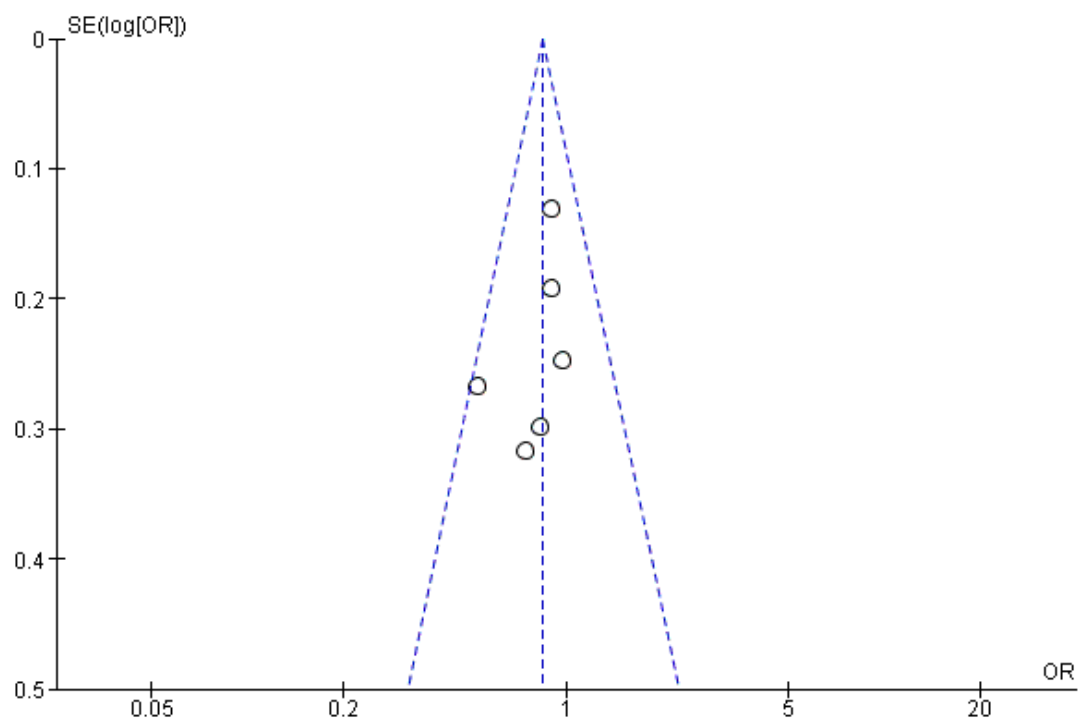

Supplement: Supplementary file 1 [file medicina-58-00491-s001.zip › Supplementary File S9.pdf]
